# Newly Identified Nucleoid-Associated-Like Protein YlxR Regulates Metabolic Gene Expression in Bacillus subtilis

**DOI:** 10.1128/mSphere.00501-18

**Published:** 2018-10-24

**Authors:** Mitsuo Ogura, Yu Kanesaki

**Affiliations:** a Institute of Oceanic Research and Development, Tokai University, Shizuoka, Japan; b Research Institute of Green Science and Technology, Shizuoka University, Shizuoka, Japan; University of Iowa

**Keywords:** RNA-Seq, metabolic genes, nucleoid, transcription regulation

## Abstract

Glucose is the most favorable carbon source for the majority of bacteria, which have several glucose-responsive gene networks. Recently, we found that in Bacillus subtilis, glucose induces expression of the extracellular sigma factor genes *sigX*/*M*. To explore the factors affecting this phenomenon, we performed a transposon mutagenesis screen for mutants with no glucose induction (GI) of *sigX-lacZ* and identified *ylxR*. YlxR is widely conserved in eubacteria. Further analysis revealed that *ylxR* is induced by glucose addition. *In vitro* DNA-binding and cytological studies suggested that YlxR is a nucleoid-associated protein (NAP) in B. subtilis. In many cases, NAPs influence transcription, recombination, and genome stability. Thus, we performed transcriptome sequencing (RNA-Seq) analysis to evaluate the impact of *ylxR* disruption on the transcriptome in the presence of glucose and observed that YlxR has a profound impact on metabolic gene expression in addition to that of four sigma factor genes. The wide fluctuations of gene expression may result in abolition of GI of *sigX*/*M* in the *ylxR* disruptant.

**IMPORTANCE** Expression of genes encoding NAPs is often temporally regulated. According to results from single-cell analysis, the *ylxR* gene is induced by glucose and expressed in a bistable mode. These characteristics have not previously been reported for NAP gene expression. Transcriptional profiling of the *ylxR* disruptant revealed a change in the expression levels of approximately 400 genes, including genes for synthesis of 12 amino acids and 4 nucleotides, in addition to the SigX/M regulons. Thus, YlxR is a critical regulator of glucose response in B. subtilis.

## Abstract (precis)

Expression of genes encoding NAPs is often temporally regulated. According to results from single-cell analysis, the *ylxR* gene is induced by glucose and expressed in a bistable mode. These characteristics have not previously been reported for NAP gene expression. Transcriptional profiling of the *ylxR* disruptant revealed a change in the expression levels of approximately 400 genes, including genes for synthesis of 12 amino acids and 4 nucleotides, in addition to the SigX/M regulons. Thus, YlxR is a critical regulator of glucose response in B. subtilis.

## INTRODUCTION

Glucose is the most favorable carbon source for the majority of bacteria, and therefore bacteria have several glucose-responsive gene networks (1). In Gram-positive bacteria, including Bacillus subtilis, the transcription factor CcpA is the master carbon catabolite regulator (1, 2). The incorporation of glucose accelerates carbon flow in glycolysis, leading to an increase of fructose 1,6-bisphosphate. This increase is thought to trigger the phosphorylation of Ser46 of HPr, a phosphocarrier protein in the sugar phosphotransferase system (P-Ser-HPr). P-Ser-HPr associates with and activates CcpA, leading to global positive and negative effects on the transcriptional network, including for genes encoding carbon metabolism enzymes. Moreover, there are several additional glucose-responsive transcription factors, such as CcpC, CcpN, CggR, and GlcT (2). In Escherichia coli, catabolite gene-activator protein (CAP [Crp]) has been considered a conventional transcription factor responding to glucose. However, recent genomic analyses led to a new idea—that CAP is a nucleoid-associated protein (3).

Bacterial chromosomal DNA had been thought to lack histones. However, bacterial histone-like proteins such as HU and IHF have been found, and their roles were clarified in phage recombination and gene transcription (4). Accumulated studies revealed that this type of proteins has the distinct nature of histones. Thus, proteins which are not structurally related to histones but have similar functions to histones have been found in bacteria. The group of proteins related to bacterial chromatin structure are called nucleoid-associated proteins (NAPs) (5). NAPs have many roles in transcription, recombination, and chromosome condensation, rearrangement, maintenance, and segregation (3). NAPs generally have DNA-binding activity, which is sequence specific and/or sequence independent, or NAPs recognize local DNA structure (3). The modes of transcriptional regulation of NAPs are diverse: for example, H-NS inhibits RNA polymerase (RNAP) progression on DNA, while Fis regulates transcription through various modes of interaction with RNAP (5). Some NAPs play roles in nutrient-dependent transcriptional regulation. For example, in E. coli, leucine-responsive regulatory protein (Lrp) regulates about 10% of all genes (6); Lrp activity is potentiated, inhibited, or unaffected by leucine for different target genes.

Recently, we found that in B. subtilis, glucose induces expression of the extracellular sigma factor genes *sigX*/*M* (7). To explore the factors affecting this phenomenon, we performed a transposon mutagenesis screen for mutants with no glucose induction (GI) of *sigX-lacZ* and identified *ylxR* (7). In this report, we confirmed the widely conserved *ylxR* gene in eubacteria as a required factor for GI of *sigX*/*M*. Further analysis revealed that *ylxR* is induced by glucose addition. *In vitro* DNA-binding and cytological studies of YlxR suggested that YlxR is a NAP in B. subtilis. Thus, we performed transcriptome sequencing (RNA-Seq) analysis to evaluate the impact of *ylxR* disruption on the B. subtilis transcriptome and observed that YlxR has a profound impact on metabolic gene expression, including on genes involved in synthesis of 12 amino acids and 4 nucleotides.

## RESULTS

### Identification of *ylxR* with no GI of *sigX-lacZ*.

**Fig. 1:**
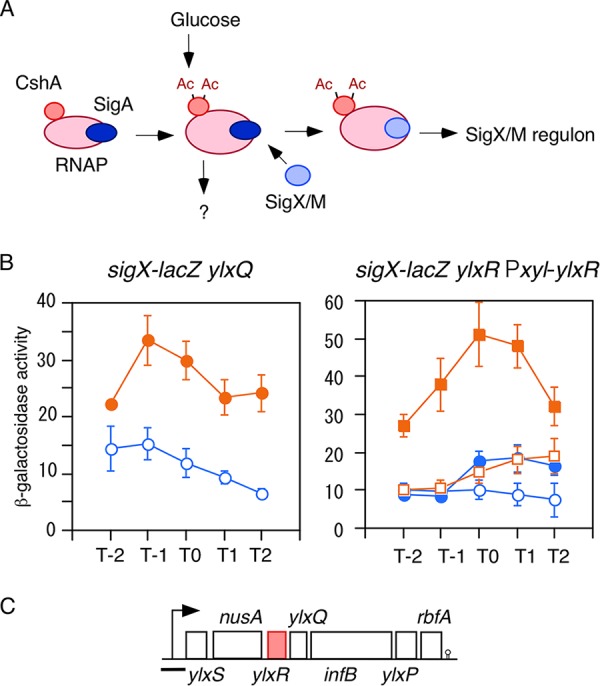
Expression of *sigX-lacZ* in a *ylxR* disruptant with artificial induction of *ylxR* and in a *ylxQ* disruptant. (A) Glucose addition stimulates the acetylation of CshA (7). CshA has been shown to associate with RNA polymerase (RNAP). RNAP with acetylated CshA may stimulate the replacement of σ^A^ by σ^X/M^ in the RNAP holoenzyme, although the mode of action is not known. σ^A^-associated RNAP holoenzyme with acetylated CshA may stimulate the transcription of some genes. Ac, acetyl moiety. (B) Cells were grown in sporulation medium with (closed symbols) or without (open symbols) 2% glucose and sampled hourly. Means from three independent experiments and the standard deviations are shown. The *x* axis represents the growth time in hours relative to the end of vegetative growth (T0). The relevant genotype is indicated above the panel. (Left) OAM765 cells. (Right) OAM736 cells. Squares and circles indicate cultures with or without 2% xylose, respectively. (C) The structure of the *ylxR*-containing operon is shown. Boxes and a bent arrow show open reading frames and the promoter, respectively. The terminator is indicated in the stem-loop form. The black bar shows the cloned promoter region used to analyze promoter expression.

In transposon mutagenesis screening for mutants with no GI of *sigX-lacZ*, we identified uncharacterized *ylxR*, as well as *cshA*, the latter encoding a DEAD box RNA helicase that has been reported to be associated with RNAP (7–9). Thus, we previously presented the model shown in Fig. 1A. Considering the structure of the *ylxR*-containing operon, Tn insertion into *ylxR* could have a polar effect on downstream genes such as *ylxQ* (Fig. 1C). Thus, a *ylxQ* disruption mutant was constructed and introduced into a strain bearing *sigX-lacZ*. Examination of β-galactosidase (β-Gal) activity in the resultant strain showed no effect of *ylxQ* disruption: i.e., GI of *sigX-lacZ* in the *ylxQ* disruptant was similar to that in the wild-type strain (Fig. 1B, left), where GI was 3-fold (7). Next, a *ylxR* disruptant with xylose-inducible *ylxR* in the *amyE* locus was constructed, and the β-Gal activity was examined. Without xylose, the strain showed a little GI, probably due to leaky expression of the xylose-inducible promoter (Fig. 1B, right). In the presence of xylose, the strain showed significant GI of *sigX-lacZ*. These results indicate that *ylxR* is involved in the GI of *sigX*. We note that the *ylxR* disruption in strain OAM735 is in fact a *ylxR* depletion mutation due to the probable leaky expression of IPTG (isopropyl-β-d-thiogalactopyranoside)-inducible Pspac-driven intact *ylxR* (see Fig. S1A in the supplemental material). The phenotype of this mutant without IPTG, however, could not be distinguished from that of the Tn-inserted *ylxR* mutant. (The depleted mutant was used in Fig. 1B, and see Fig. 6B below, where the depleted and Tn mutants are compared.) Thus, we consider both to be similar mutants.

10.1128/mSphere.00501-18.1 FIG S1 Expression of P*ylxS* under various conditions. The strains were grown in sporulation medium or medium supplemented with glucose, Tris-HCl, or xylose. β-Galactosidase activities are shown in Miller units. Means from three to five independent experiments and the standard deviations are shown. The *x* axis represents the growth time in hours relative to the end of vegetative growth (T0). (A) Expression in medium containing various concentrations of glucose (open circles, 0%; closed circles, 0.1%; closed triangles, 1%; closed inverted triangles, 2%) in OAM741. Along the panel, the chromosomal structure of OAM735 is shown. (Symbols are explained in the legend to Fig. 1.) (B) (Left) Expression of P*ylxS*-*lacZ* (OAM741 in medium containing 25 mM Tris-HCl [pH 7.5]). Open and closed squares show growth without and with 2% glucose, respectively. (Right) Changes of ambient pH. Open and closed circles show nonbuffered growth without and with 2% glucose, respectively. Open and closed squares show growth in medium supplemented with Tris-HCl without and with 2% glucose. (C) OAM741 (wild type [circles]) and OAM769 (*pdhC* [squares]) cells. Closed and open symbols show growth with and without 2% glucose, respectively. (D) Strains were grown in medium with (closed symbols) or without (open symbols) 2% glucose in the presence of 1% xylose. Circles, OAM743 (*cshA* disruption and xylose-inducible P*xyl-cshA* [wild type]). Squares, OAM814 (*cshA* disruption and xylose-inducible P*xyl-cshA* [K-to-R mutant]). Download FIG S1, PDF file, 0.5 MB. Copyright © 2018 Ogura and Kanesaki. 2018 Ogura and Kanesaki This content is distributed under the terms of the Creative Commons Attribution 4.0 International license.

The *ylxR* gene encodes a small, basic protein (molecular weight [MW], 10.3 kDa; isoelectric point, 10.09) with uncharacterized domain DUF448, and it is widely conserved in eubacteria (see Fig. S2 in the supplemental material). According to the Pfam database, 2,052 species have a YlxR ortholog. The structure of Streptococcus pneumoniae YlxR has been resolved, and it is proposed to be able to bind nucleic acids (10). In the B. subtilis genome, *ylxR* is associated with the essential genes *nusA* and *infB*, respectively, encoding transcription terminating factor and translation initiation factor B in a single mRNA (11, 12).

10.1128/mSphere.00501-18.2 FIG S2 Phylogenetic tree of YlxR in bacteria. The tree was generated by use of CLC sequence viewer 8 (neighbor-joining method, bootstrap analysis of 1,000 replicates). The scale bar indicates the number of amino acid substitutions per site. Download FIG S2, PDF file, 1.1 MB. Copyright © 2018 Ogura and Kanesaki. 2018 Ogura and Kanesaki This content is distributed under the terms of the Creative Commons Attribution 4.0 International license.

### GI of P*ylxS* by CshA.

**Fig. 2:**
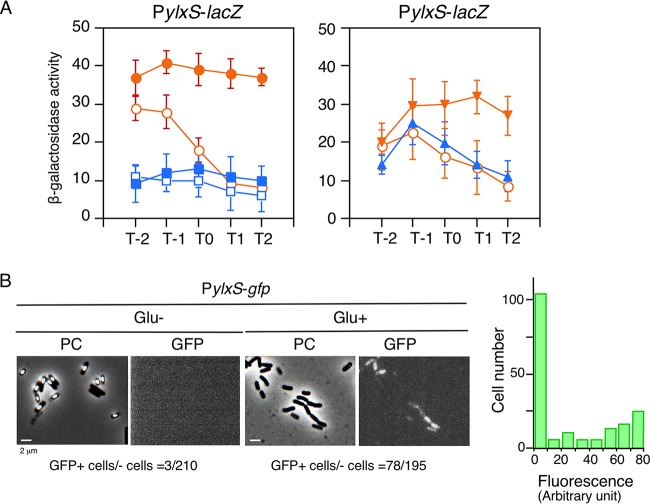
Expression of *ylxR*-containing operon revealed by *lacZ* and *gfp* fusion analysis. (A) β-Galactosidase activities from samples taken hourly are shown in Miller units. Means from three independent experiments and standard deviations are shown. The *x* axis represents the growth time in hours relative to the end of vegetative growth (T0). (Left) Cells were grown in sporulation medium with (closed symbols) or without (open symbols) 2% glucose. Circles and squares indicate the wild-type (OAM741) and *cshA* disruption mutant (OAM742), respectively. (Right) OAM741 cells were grown in sporulation medium with 3% sodium succinate (closed triangles) or 1% glycerol (closed inverted triangles) or without additional carbon sources (open circles). (B) Strain OAM818 (P*ylxS-gfp*) was grown in sporulation medium with or without 2% glucose. After 14 h, cells were sampled and processed. Microscopic observation is shown. PC, phase contrast; GFP, green fluorescent protein. A histogram of fluorescence intensities of the cells with glucose is shown alongside the panel. GFP fluorescence was visualized using the NIBA filter set (Olympus). Image processing and data analysis were performed using Adobe Photoshop CS5.

As *sigX*/*M* expression is induced by glucose, it was expected that *ylxR* expression driven by a major sigma factor, SigA, is also induced by glucose. Indeed, we observed that the P*ylxS* promoter, which drives the transcription of *ylxR*, was induced by glucose (Fig. 2A, left). This GI was not dependent on glucose concentration (0.1 to 2%), as shown in Fig. S1A. A previous report showed that addition of glucose and glutamine to sporulation medium repressed expression of several genes regulated by the transition state regulators SigH and AbrB (13). The addition of glucose resulted in “overflow” metabolism, leading to a decrease in external pH. Buffering of this pH decrease by addition of Tris-HCl (pH 7.5) rescued the repression, which means external pH controls SigH/AbrB-mediated gene expression. Thus, we tested a similar possibility with respect to the mechanism of GI of P*ylxS*: i.e., whether buffering the decrease in pH by addition of Tris-HCl (pH 7.5) affected the GI. We observed that glucose addition resulted in a pH decrease, and addition of Tris-HCl (pH 7.5) significantly buffered this pH decrease, as expected (Fig. S1B, right). However, we still observed GI of P*ylxS* when the external pH was buffered (Fig. S1B, left), excluding external pH control of gene expression as a mechanism for this GI, probably because no glutamine was added. We note that in the *ylxR* disruptant, external pH similarly decreased to that in the wild-type strain, when glucose was added to the medium (data not shown).

In the *cshA* mutant, GI of *sigX* was abolished; thus, it is possible that *cshA* disruption may also abolish GI of P*ylxS* (7) (Fig. 1A). In fact, when *cshA* was disrupted, the basal transcription levels of P*ylxS* were reduced, and no GI was observed (Fig. 2B, left). Proteomic analysis of B. subtilis revealed that CshA, a DEAD box helicase, is acetylated at K244 and K296 (14). Protein acetylation often modifies protein function (15). We recently found acetylated CshA-dependent GI of genes for SigX and SigM (7). The GI of P*ylxS* may also require CshA acetylation, like *sigX*/*M*, as the GI of P*ylxS* was abolished in a *pdhC* (encoding a subunit of pyruvate dehydrogenase) disruptant where acetyl coenzyme A (acetyl-CoA) would be depleted (Fig. S1C).

We then tested the effect of addition of two carbon sources to the medium—glycerol and succinate—at an equal molar concentration of glucose. The former is incorporated into glycolysis and the latter into the tricarboxylic acid (TCA) cycle (2). We observed induction of P*ylxS* by glycerol, but not by succinate (Fig. 2A, right). This observation is also consistent with the above hypothesis involving CshA acetylation, because the addition of glycerol but not succinate leads to increase in acetyl-CoA. Moreover, addition of 3% succinate had no effect on the P*ylxS* expression, and 0.1% glucose induced this promoter (Fig. S1A), excluding the possibility that high osmotic pressure caused the GI of P*ylxS*.

To test the acetylation hypothesis, we constructed a strain in which native *cshA* was disrupted but artificial induction of wild-type *cshA* was feasible from a xylose-inducible promoter. First we observed that *cshA* induction by addition of xylose complemented the expression and GI of P*ylxS* in the *cshA* disruptant (Fig. S1D). Induction of a mutant form of *cshA* with K-to-R substitutions at two acetylated lysine residues did not complement the GI of P*ylxS*. We made His-tagged versions of the wild type and the K-to-R mutant in B. subtilis and purified the proteins: similar amounts of the proteins were obtained, suggesting that the wild-type and mutant proteins had similar stability (data not shown). Collectively, these observations strongly suggest a requirement for CshA acetylation for the GI of P*ylxS*.

### GI of P*ylxS* among the cell population.

To observe GI of P*ylxS* at the single-cell level, we constructed a transcriptional P*ylxS*-*gfp* fusion and performed microscopic analysis. Since even in the presence of glucose, fluorescence of green fluorescent protein (GFP) was very low and observed from a part of the cell population in log phase (data not shown), the cells were observed in stationary phase. Sporulation is known to be under catabolite repression (16), and thus, only in the phase-contrast images of cells grown without glucose was the progress of spore formation observed (Fig. 2B). Among cells grown without glucose, GFP-positive cells were scarcely observed, while among cells grown with glucose, one-third were GFP positive. Based on these results, we concluded that under the conditions tested, glucose induced the expression of P*ylxS* in a bistable mode, where the distribution of fluorescence is bimodal. Since P*ylxS* drives transcription of two essential genes, it is possible that basal expression of P*ylxS* occurs in cells without glucose. In fact, such basal expression was observed through the P*ylxS*-*lacZ* fusion in the absence of glucose; using the P*ylxS*-*gfp* fusion, it was also observed in nearly all cells in Luria-Bertani (LB) medium (see Fig. S3 in the supplemental material). We note that in a former report where a NusA-GFP translational fusion was analyzed, fluorescence seemed to be homogeneously detected from all the cells observed (17). The cause of this difference is not known; however, some experimental differences can be pointed out, such as the media used and the fusion construction strategy.

10.1128/mSphere.00501-18.3 FIG S3 Microscopic observation of P*ylxS*-*gfp*. OAM818 cells were grown in Luria-Bertani medium. After 14 h, cells were sampled and processed as described in Materials and Methods. Microscopic observation is shown. PC, phase contrast; GFP, green fluorescent protein. Download FIG S3, PDF file, 0.5 MB. Copyright © 2018 Ogura and Kanesaki. 2018 Ogura and Kanesaki This content is distributed under the terms of the Creative Commons Attribution 4.0 International license.

### NAP-like protein YlxR.

**Fig. 3:**
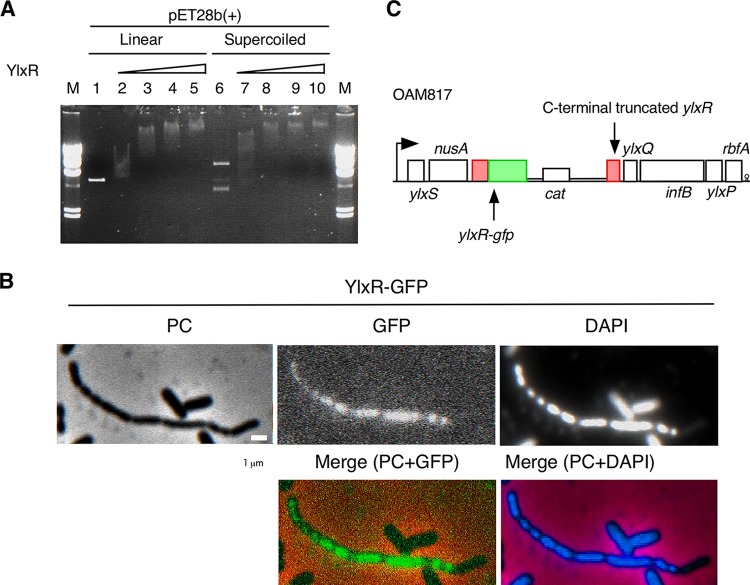
Electrophoretic mobility shift assay using purified YlxR and localization analysis of YlxR-GFP. (A) Purified YlxR was increasingly added to the indicated DNA template [20 ng of pET28b(+)], and its electrophoretic mobility shift was determined in a 1% agarose gel. In lanes 1 and 6, 2 and 7, 3 and 8, 4 and 9, and 5 and 10, there were 0, 100, 200, 400, and 800 nM YlxR, respectively. Lane M contains the marker (Lambda/HindIII). (B) Strain OAM817 (*ylxR*::*gfp*) was grown in sporulation medium with 2% glucose. After 14 h, cells were sampled and processed. Microscopic observation is shown. PC, phase contrast; GFP, green fluorescent protein; DAPI, 4′,6-diamidino-2-phenylindole. Fluorescence of GFP and DAPI was visualized using WIB and WU filter sets (Olympus), respectively. Image processing and data analysis were performed using Adobe Photoshop CS5. Representative images are shown. Indicated merged photos are in pseudocolor. (C) The chromosomal structure of OAM817 is depicted (symbols as in Fig. 1).

The structure of YlxR has been resolved and suggested its probable DNA/RNA-binding activity; however, this has not been experimentally verified (10). To test this possibility, we purified intact YlxR and then applied it to electrophoretic mobility shift assay (EMSA). As shown in Fig. 3A, YlxR was able to bind to the pET28b(+) vector, suggesting that the DNA-binding activity of YlxR has low sequence specificity. YlxR binding slightly preferred the supercoiled form of this vector DNA compared to its linear form (compare lane 2 to lane 7 in Fig. 3A).

YlxR is a small basic protein with DNA-binding activity that shows low levels of sequence dependency, which suggested that YlxR shares certain characteristics with some NAPs (3). To examine whether YlxR is associated with nucleoids in the cell, we constructed *ylxR-gfp*, where *gfp* is fused to the C terminus of *ylxR*, and observed its localization. Cells grown with glucose underwent microscopic observation, and the fluorescence derived from YlxR-GFP seemed to be associated with nucleoids, as was observed for Rok and other proteins associated with the nucleoid in B. subtilis (18, 19) (Fig. 3B). The strain with the fusion at the original locus (Fig. 3C) grew in synthetic minimal medium (data not shown [see Fig. 6B]), suggesting that YlxR-GFP would be functional. We observed heterogeneous distribution of YlxR-GFP among the cells, as well as in the case of P*ylxS-gfp* (Fig. 3B and data not shown). Thus, we consider YlxR to be a NAP-like protein. We note that the apparent observed dissociation constant of YlxR for DNA (about 100 nM) is within the range reported for E. coli NAPs (20).

### Transcriptome analysis of *ylxR* disruptant in the presence of glucose.

**Fig. 4:**
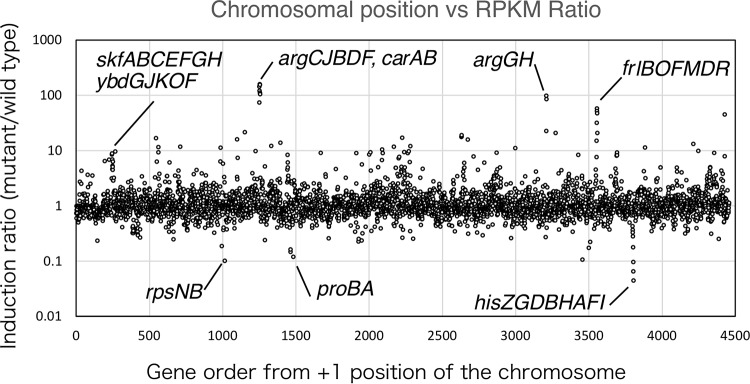
Operons whose transcript levels were changed significantly by *ylxR* disruption. Values of fold change of transcripts between wild-type and *ylxR* mutant cells were calculated from three independent RNA-Seq analyses. All genes (ordered clockwise from the +1 position of the chromosome) are plotted against fold change values.

It is known that many NAPs affect the transcriptome (3, 5). To test this possibility for YlxR, we performed RNA-Seq analysis of the *ylxR* disruptant in the presence of glucose using the wild-type strain as the reference with three biological replicates of early-stationary-phase cells. Expression of 128 and 265 genes was, respectively, downregulated (<1/2) and upregulated (>2.0) (Fig. 4; see Table S1 in the supplemental material).

10.1128/mSphere.00501-18.5 TABLE S1 List of differentially expressed genes in *ylxR*. Download Table S1, PDF file, 0.5 MB. Copyright © 2018 Ogura and Kanesaki. 2018 Ogura and Kanesaki This content is distributed under the terms of the Creative Commons Attribution 4.0 International license.

### Sporulation-related genes.

Spo0A is a master regulator of sporulation initiation (21). In the *ylxR* disruptant, the expression of 10 Spo0A-activated transcription units (22 genes) was upregulated (from *skfB* at 8.9-fold to *putB* at 2.1-fold [see Table S2a in the supplemental material]). In the presence of glucose, YlxR represses the expression of two Spo0A-activated sigma genes required for sporulation initiation (*sigE* and *sigF*). It should be noted that increased SigE and SigF regulon expression in the *ylxR* disruptant leads to increased SigK and SigG regulon expression due to the so-called “sigma cascade” (21). Thus, the *ylxR* gene may be involved in the catabolite repression of sporulation.

10.1128/mSphere.00501-18.6 TABLE S2 (a) Spo0A-activated genes and genes that are not driven by sigma A, sigma H, and sigma B among the differentially expressed genes in the *ylxR* disruptant with glucose. (b) Genes involved in cofactor synthesis among the differentially expressed genes in the *ylxR* disruptant with glucose. Download Table S2, PDF file, 0.1 MB. Copyright © 2018 Ogura and Kanesaki. 2018 Ogura and Kanesaki This content is distributed under the terms of the Creative Commons Attribution 4.0 International license.

### Sigma regulons.

Initial Tn mutagenesis showed that in the *ylxR* disruptant in the presence of glucose, the expression of *sigX*/*M* genes decreased. As expected, the expression of some SigX/M regulon genes was decreased (Table S2a). The RNA-Seq analysis showed that expression of the motility-related SigD regulon and nitrogen starvation-regulated SigL-regulon increased in the *ylxR* disruptant in the presence of glucose without increased expression of the *sigD* and *sigL* genes themselves (Table S2a) (22). This may be due to some changes in the competition status of RNAP for sigma factors, from the point of view of the so-called “sigma cycle” (23). The changes would be caused by enhanced expression of *sigE* and *sigF* and reduced expression of *sigM*. (For an unknown reason, the expected decrease of *sigX* expression was not detected in the RNA-Seq analysis.)

### Metabolic genes.

**Fig. 5:**
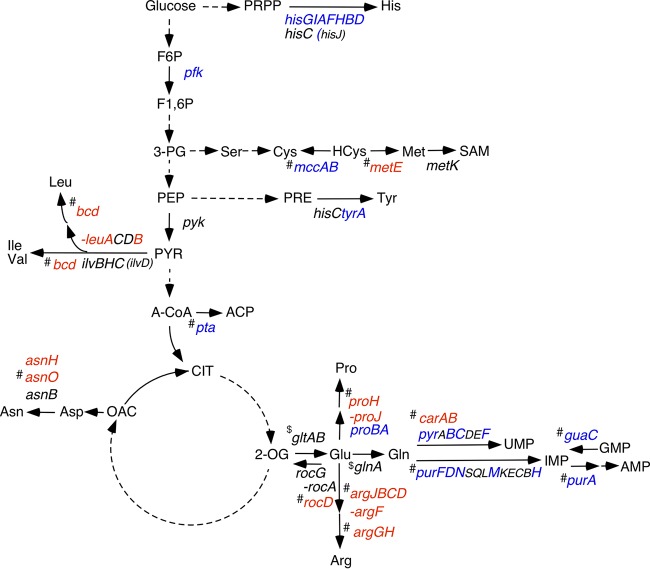
Fluctuation of metabolic gene expression in the *ylxR* disruptant with glucose. Metabolic pathways, including glycolysis, the Krebs cycle, and synthesis of amino acids and nucleotides, are shown. Solid arrows show reactions catalyzed by enzymes encoded by the indicated genes, and dotted arrows show multistep reactions. Genes whose expression decreased or increased in the RNA-Seq analysis of the *ylxR* strain in the presence of glucose are shown in blue and red, respectively. Genes in black in parentheses are those whose expression was not changed in RNA-Seq analysis; however, genes whose expression was not significantly changed in RNA-Seq but changed in *lacZ* analysis of the promoter for the gene are shown in black without parentheses. Genes not tested with *lacZ* analysis, but whose expression should be changed due to the gene position in the operon, are included in this category. Genes presented as consecutive characters indicate operons. # and $ indicate genes whose expression was not analyzed by *lacZ* analysis and were assigned to opposite categories (i.e., both up- and downregulation) in three replicates of RNA-Seq. F6P, fructose-6-phosphate; F1,6P, fructose-1,6-biphosphate; 3-PG, 3-phosphoglycerate; PEP, phosphoenolpyruvate; PYR, pyruvate; A-CoA, acetyl-CoA; ACP, acetylphosphate; PRPP, phosphoribosyl pyrophosphate; HCys, homocysteine; SAM, *S*-adenosyl methionine; PRE, prephenate; CIT, citric acid; 2-OG, 2-oxoglutarate; OAC, oxaloacetate.

**Fig. 6:**
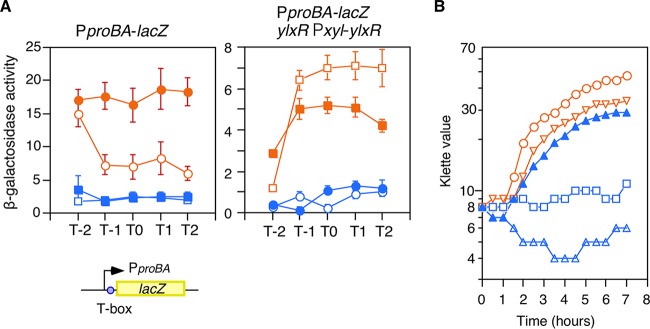
Effects of *ylxR* disruption on *proBA* expression and cell growth in minimal medium. (A) β-Galactosidase activities from samples taken hourly are shown in Miller units. Means from three independent experiments and standard deviations are shown. The *x* axis represents the growth time in hours relative to the end of vegetative growth (T0). (Left) Cells were grown in sporulation medium with (closed symbols) or without (open symbols) 2% glucose and sampled hourly. Circles and squares indicate the wild-type (OAM821) and *ylxR* disruptant (OAM822), respectively. The chromosomal structure of the fusion is depicted below the data (symbols as in Fig. 1). (Right) OAM841 cells were grown in sporulation medium with (closed symbols) or without (open symbols) 2% glucose. Squares and circles indicate culture with or without 2% xylose, respectively. (B) Growth curves in synthetic minimal medium. Overnight culture of each strain in one-step competence medium was inoculated into 4 ml of minimal medium in an L-tube, and then growth was monitored with a Klett calorimeter (Fisher Scientific, Waltham, MA). Representative results are shown. Symbols: B. subtilis 168, circles; OAM735 (*ylxR*-depleted mutant), squares; OAM816 (*ylxR*::Tn), triangles; OAM840 (*ylxQ* disruptant), inverted triangles. Solid triangles indicate OAM816 supplemented with proline (10 μg/ml).

**Fig. 7:**
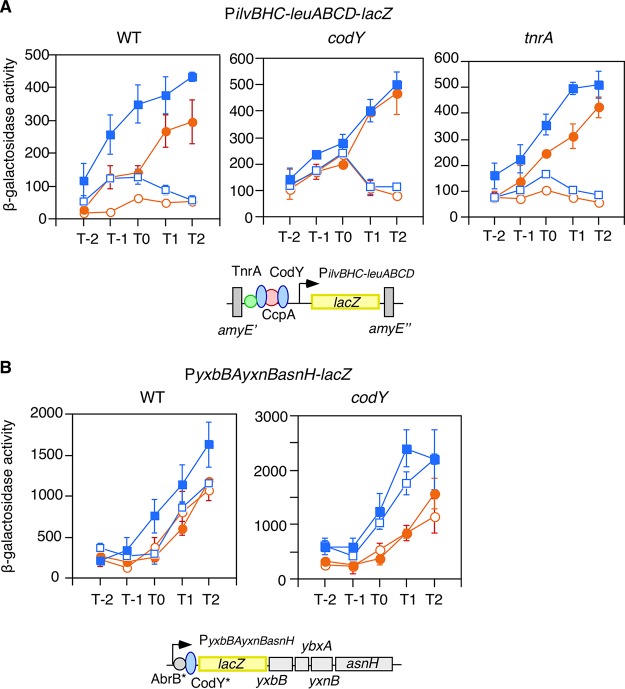
Expression analysis of the *ilvB-leu* and *asnH*-containing operons by *lacZ* fusions. Cells were grown in sporulation medium with (closed symbols) or without (open symbols) 2% glucose and sampled hourly. Circles and squares indicate the wild-type and *ylxR* disruptant, respectively. β-Galactosidase activities are shown in Miller units. Means from three independent experiments and standard deviations are shown. The *x* axis represents the growth time in hours relative to the end of vegetative growth (T0). The fusions tested and the relevant genotypes are indicated. The chromosomal structures of the fusions are depicted below the data (symbols as in Fig. 1). Various transcription factors are indicated by circles and ovals, whose binding sites on the promoters are depicted according to SubtiWiki (63). An asterisk indicates that the correct binding site of the transcription factor has not been reported. (A) (Left) FU676 and OAM820. (Middle) OAM843 and OAM845. (Right) OAM844 and OAM846. (B) (Left) YXBBd and OAM849. (Right) OAM847 and OAM848.

RNA-Seq analysis and subsequent LacZ analysis revealed differentially expressed genes involved in the synthesis of 12 amino acids (Arg, Asn, Cys, Glu, Gln, His, Ile, Leu, Met, Pro, Tyr, and Val) and 4 nucleotides (UMP, GMP, IMP, and AMP) in the *ylxR* disruptant in the presence of glucose (Fig. 5 to 7; see Fig. S4 in the supplemental material). Thus, YlxR may be a regulator for adaptation to the new metabolic state caused by glucose addition, especially of nitrogen metabolism, including biosynthesis/degradation of amino acids. Expression of several genes was confirmed using *lacZ* fusions. We newly identified GI or glucose repression (GR) of some genes (Fig. 6 and 7; Fig. S4). The expression of P*proBA* is positively regulated by YlxR irrespective of glucose addition, because *ylxR* disruption severely reduced *proBA* expression (Fig. 6A, left). This decrease in *proBA* expression in the *ylxR* disruptant was complemented by artificial expression of *ylxR* from P*xyl*, demonstrating the role of *ylxR* in the *proBA* expression (Fig. 6A, right). We had observed previously that *proBA* disruption led to proline auxotrophy (24); thus, it was expected that the *ylxR* disruptant would be unable to grow in synthetic minimal medium. This was the case, and the observation that the *ylxQ* disruptant was able to grow in this medium reinforced the role of *ylxR* in growth, but not the involvement of the downstream gene *ylxQ* (Fig. 6B). Proline addition restored the growth of the *ylxR* disruptant, which is consistent with the proline auxotrophy of the strain. We note that *proJ* expression increased in the *ylxR* mutant with glucose (Fig. 5); however, *proJ* expression was very weak (about 5% of that of *proBA* in the *ylxR* disruptant with glucose).

10.1128/mSphere.00501-18.4 FIG S4 Expression analysis of metabolic genes determined using *lacZ* fusions. Cells were grown in sporulation medium with (closed symbols) or without (open symbols) 2% glucose and sampled hourly. Circles and squares indicate the wild-type and *ylxR* disruptant, respectively. β-Galactosidase activities are shown in Miller units. Means from three independent experiments and standard deviations are shown. The *x* axis represents the growth time in hours relative to the end of vegetative growth (T0). The fusions tested are indicated. The chromosomal structure of the fusions is shown below the data (symbols as in Fig. 1). Various transcription factors are indicated by circles and ovals, whose binding sites on the promoters are depicted according to SubtiWiki (63). The deduced YlxR function on each gene is indicated above the data. GI, glucose induction; GR, glucose repression. GI or GR in bold letters was newly found in this study. (A) OAM819 and OAM851. (B) OAM850 and OAM831. (C) OAM832 and OAM833. (D) OAM834 and OAM835. (E) OAM827 and OAM828. (F) OAM838 and OAM839. (G) BFS56 and OAM823. (H) OAM825 and OAM826. To construct pIS-*glnR*, pIS-*pfk*, pIS-*gltA*, pIS-*hisZ*, and pIS-*pyrR*, PCR products amplified from chromosomal DNA using the oligonucleotide pairs pIS-glnR-E/pIS-glnR-B, pIS-pfk-F(E)/pIS-pfk-R-(B), pIS-gltA-E/pIS-gltA-B, pIS-hisZ-E/pIS-hisZ-B, and pIS-pyrR-F(E)/pIS-pyrR-R(B), respectively, were digested with EcoRI/BamHI and cloned into pIS284 treated with the same enzymes (54). To construct pMutin-*rocA* and pMutin-*tyrA*, PCR products amplified from chromosomal DNA using the oligonucleotide pairs Mut-rocA-E/Mut-rocA-B and tyrA-Mut-E/ptyrA-Mut-B, respectively, were digested with EcoRI/BamHI and cloned into pMutin2 treated with the same enzymes (60). Download FIG S4, PDF file, 0.9 MB. Copyright © 2018 Ogura and Kanesaki. 2018 Ogura and Kanesaki This content is distributed under the terms of the Creative Commons Attribution 4.0 International license.

The *ilvBHC-leuABCD* operon, whose products are involved in synthesis of branched-chain amino acids, is regulated by the catabolite control protein A (CcpA) and CodY/TnrA, which sense branched-chain amino acid/nitrogen availability, respectively (25, 26). Expression of this operon is induced by glucose in a CcpA-dependent manner, and *ylxR* disruption further increased the expression irrespective of glucose addition (Fig. 7A, left). YlxR may function at P*ilvB* cooperatively with CodY and TnrA. Thus, we examined the effect of *ylxR* disruption on this operon expression in *codY* or *tnrA* disruptants. In the *tnrA* disruptant, the enhancing effect of *ylxR* disruption was clearly observed, while in the *codY* disruptant, no effect of *ylxR* disruption was observed (Fig. 7A, middle and right). These results suggest that YlxR functions at this promoter in a CodY-dependent manner. YlxR regulates about 10% of the total genes in B. subtilis (Table S1), but 27% of the CodY regulon (14/51 operons) is regulated by YlxR, which is a notable difference in proportion (Table S1). However, only 16% of the TnrA regulon (6/37 operons) is regulated by YlxR. In a *ccpA* background, basal P*ilvB* expression was very low (around 8 Miller units), but a significant effect of *ylxR* disruption was observed (data not shown). This might be consistent with the observation that 10% of the CcpA regulon transcription units belong to the YlxR regulon, suggesting no specific relationship between CcpA and YlxR.

Next, we observed that expression of the *asnH*-containing operon was significantly enhanced in the *ylxR* disruptant only in the presence of glucose, indicating *ylxR* negatively regulates this operon (Fig. 7B, left). The promoter of the *asnH*-containing operon is also known to be bound by CodY (27). Thus, we explored the possibility that at this promoter, YlxR may require CodY for its function. In a *codY* disruptant, expression of this operon was similarly enhanced by the introduction of the *ylxR* disruption both with and without glucose (Fig. 7B, right). Moreover, the enhancement ratio by the *ylxR* disruption was larger than that in the *codY*^+^ cells. Thus, *codY* disruption enhanced YlxR function, suggesting that CodY may weaken YlxR function. This role of CodY is different from the case in the *ilv-leu* operon, where YlxR could enhance CodY function, suggesting that YlxR works with CodY in a context-dependent manner. We note no regulatory relationship between transcription of *codY* and *ylxR* with each other (Table S1 and data not shown). It has been reported that there are three genes encoding asparagine synthesis enzymes in B. subtilis (28). Thus, we examined *asnB* expression in RNA-Seq and found that the decrease of *asnB* expression in the *ylxR* disruptant was on the threshold of being a significant change in gene expression (0.53-fold [ratio of the expression levels in the *ylxR* disruptant versus strain 168; designated here “*ylxR*/168”]). We confirmed substantial levels of decrease of fusion expression by using P*asnB*-*lacZ* (about 0.5-fold [Fig. S4G]). Interestingly, the *asnB* gene is cotranscribed with the upstream gene *metK*, which is involved in methionine metabolism (Fig. 5).

*gltAB* encode glutamate synthetase: among replicate RNA samples, different results were obtained for *gltA* (0.49-, 1.17-, and 5.02-fold [*ylxR*/168]) and *gltB* (0.15-, 1.73-, and 6.56-fold [*ylxR*/168]), respectively. To measure actual gene expression in the *ylxR* disruptant, we constructed a P*gltAB*-*lacZ* fusion and examined its β-Gal activity (Fig. S4E). Expression of the fusion was significantly decreased in the *ylxR* disruptant in the presence of glucose, indicating that YlxR positively regulates P*gltAB*. P*gltAB* is negatively regulated by glutamine-bound RocG (glutamate dehydrogenase) through sequestration of the positive transcription factor GltC (29) by RocG. *rocGA* expression is negatively regulated by CcpA in the presence of glucose (29). Thus, glucose addition finally results in the enhancement of P*gltAB* activity. *rocGA* is a candidate gene regulated by YlxR because the operon expression was reduced in RNA-Seq analysis, although the effect was not statistically significant. The disruption of *ylxR* counteracted glucose repression of P*rocGA* to some extent in *lacZ* analysis, leading to abolition of GI of P*gltAB* (Fig. S4A and E). According to the RNA-Seq analysis, the mRNA abundance of *glnRA* was also inconsistent among replicates (for *glnA*, 0.41-, 2.07-, and 1.24-fold [*ylxR*/168]). Thus, we undertook β-Gal analysis of P*glnRA*-*lacZ* (Fig. S4B). In the *ylxR* disruptant, P*glnRA* expression decreased in the presence of glucose.

The expression of the six-gene operon for glycolysis *cggR-gapA-pgk-tpi-pgm-eno* is repressed by CggR, and glucose addition resulted in GI of these genes (30). Other glycolysis genes include the *pfk-pyk* operon, whose expression was mildly induced by glucose (31), and we confirmed GI of the *pfk-pyk* operon (Fig. S4D). Moreover, we observed that *ylxR* disruption significantly weakened this GI, suggesting the involvement of YlxR in regulation of this operon. Furthermore, *ylxR* disruption increased the expression of five genes encoding redox enzymes in the electron-transport system (*ctaEDFG* and *qcrB*) (Table S1). Finally, *ylxR* disruption affected five genes involved in the synthesis of four cofactors (Table S2b).

## DISCUSSION

The initial goal in this study was to identify causes for GI of *sigX*/*M*. Glucose-induced YlxR would change the competition status of several sigma factors for binding to the RNAP core enzyme, maybe leading to abolition of GI of *sigX/M*. However, more interestingly, we identified a new glucose-responsive system that includes protein lysine acetylation of CshA (Fig. 1A; Fig. S1D), bistable expression of NAP-like protein YlxR (Fig. 2B), and transcriptional regulation of metabolic genes by YlxR (Fig. 5). Some NAPs have a role in nutrient-responsive transcription regulatory networks (3). Thus, this work enriches knowledge about such NAPs, by showing that YlxR plays a role in a glucose-responsive transcription network. We observed that the effects of the *ylxR* disruption were lost or strengthened in the *codY* disruptant, depending on the target gene, suggesting some relationship or interaction between YlxR and CodY at the promoter region of relevant genes.

Single-cell analysis of P*ylxS*-*gfp* revealed the bistable mode of its expression: i.e., in some cells GFP was produced, while in the other cells, little or no GFP was produced. Typically, the bistable expression of genes is generated by positive-feedback regulation (32). In the case of P*ylxS*, NusA-dependent negative-feedback regulation was reported (12), which, however, does not generate bistable expression. Glucose-induced and *ylxR*-dependent expression of some metabolic genes was observed, such as *gltAB*, *pfk-pyk*, and *proBA*. Recently, the customary view of metabolic gene expression as homogeneous has been challenged because bistable or heterogeneous metabolic gene expression has been reported (33–35). Thus, it is an interesting question whether the observed enhanced expression is universal or heterogeneous within the cell population under glucose-rich conditions due to the bistable expression of *ylxR*.

Several NAPs have been identified in B. subtilis (HBsu, LrpC, and Rok). However, knowledge of the impact of the two former proteins on the transcriptome is not comprehensive (18, 36–38). HBsu and LrpC regulate their own genes (39, 40). Rok regulates the competence master regulator gene *comK* directly and the biofilm-related *bslA* gene indirectly (41, 42), in addition to other genes, including the mobile and foreign genetic element genes in ICEbs1. However, Rok only regulates 39 genes (41). In contrast, YlxR has a profound impact on the B. subtilis transcriptome, although the YlxR-regulated gene list contains genes regulated by direct DNA binding and through indirect effects. We note that YlxR downregulates many SPbeta phage genes like Rok does ICEbs1 (23 genes in Table S1). However, many such genes are missing from Table S1 since the levels of the phage gene expression were very low, leading to large fluctuations in expression and resultant high *P* values in our analysis. YlxR is widely conserved in eubacteria—for example, in species belonging to the most deeply rooted phylum, Aquificae (Desulfobacteraceae in Fig. S2). Thus, it will be interesting to determine whether YlxR also functions as an NAP regulating metabolic gene expression in other eubacteria.

YlxR affects regulation of many metabolic genes, especially in the presence of glucose. However, this does not straightforwardly lead to YlxR-mediated changes of the cellular metabolome in response to glucose-rich conditions. After the transcription of genes encoding metabolic enzymes, the corresponding mRNA must be translated into protein, which may be further modified, for example, by phosphate and/or acetyl moieties (43–47). Moreover, catalytic activity of enzymes is regulated by allosteric binding of end products and/or *in vivo* substrate concentrations in addition to posttranslational modifications. However, in the transition between carbon sources (glucose and malate), changes of transcription levels of >2,000 genes were observed (48). YlxR may play a critical role in such changes because of the observed large changes in the transcriptome of the *ylxR* disruptant. In addition, metabolic gene regulation is an important factor determining cellular metabolic state, because metabolic gene regulation underlies the rapidly changing metabolome that responds to extracellular environments, including nutritional status. Thus, YlxR is an important factor for adaptation of B. subtilis cells to a glucose-rich environment.

## MATERIALS AND METHODS

### Strains and media.

All B. subtilis strains used in this study are listed in Table 1 and in Table S3 in the supplemental material. One-step competence medium (MC) (49), Schaeffer’s sporulation medium (SM) (16), LB medium (Difco, Lennox), and Spizizen’s minimal medium (50) were used. Antibiotic concentrations were described previously (51, 52). Synthetic oligonucleotides were commercially prepared by Tsukuba Oligo Service (Ibaraki, Japan) and are listed in Table S4 in the supplemental material.

**TABLE 1:** Strains and plasmids used in this study

Strain or plasmid	Strain genotype or plasmid description^*a*^	Reference or source
Strains		
168	*trpC2*	Laboratory stock
YlxRd	*trpC2 ylxR*(Em^r^ *lacZ*)	60 (BSORF)
OAM735	*trpC2 ylxR*(Em^r^ *lacZ*::Tc^r^)	This study
OAM816	*trpC2 ylxR*::Tn (inserted into 51st codon of *ylxR* ORF, Km^r^)	This study
OAM840	*trpC2 ylxQ*(Tc^r^)	This study
OAM722	*trpC2 cshA*(Tc^r^)	7
OAM709	*trpC2 thrC*::*sigX-lacZ*(Em^r^)	7
OAM736	*trpC2 thrC*::*sigX-lacZ*(Em^r^) *amyE*::P*xyl-ylxR*(Cm^r^) *ylxR*(Em^r^ *lacZ*::Tc^r^)	This study
OAM765	*trpC2 thrC*::*sigX-lacZ*(Em^r^) *ylxQ*(Tc^r^)	This study
OAM741	*trpC2 thrC*::P*ylxS-lacZ*(−284/+77, Sp^r^)	This study
OAM742	*trpC2 thrC*::P*ylxS-lacZ*(−284/+77, Sp^r^) *cshA*(Tc^r^)	This study
OAM817	*trpC2 ylxR*::*gfp*(Cm^r^)	This study
OAM818	*trpC2 amyE*::P*ylxS*-*gfp*(−284/+77, Cm^r^)	This study
OAM-N41	*amyE*::P*tapA*-*gfp*(Cm^r^::Tc^r^)	53
OAM-829	*trpC2 amyE*::P*trmK*-*lacZ*(Cm^r^)	This study
OAM-830	*trpC2 amyE*::Pt*rmK*-*lacZ*(Cm^r^) *ylxR*(Em^r^ *lacZ*::Tc^r^)	This study
OAM821	*trpC2 proB*::pSPB106(Tc^r^)	24 (original background, CU741)
OAM822	*trpC2 proB*::pSPB106(Tc^r^) *ylxR*(Km^r^)	This study
OAM841	*trpC2 proB*::pSPB106(Tc^r^) *ylxR*(Km^r^) *amyE*::P*xyl-ylxR*(Cm^r^)	This study
FU676	*trpC2 amyE*::P*ilvB*-*lacZ*(−284/+26, Cm^r^)	26
OAM820	*trpC2 amyE*::P*ilvB*-*lacZ*(−248/+26, Cm^r^) *ylxR*(Km^r^)	This study
OAM842	*trpC2 amyE*::P*ilvB*-*lacZ*(−248/+26, Cm^r^::Tc^r^)	This study
KK21	*trpC2 codY*(Cm^r^)	61
KK97	*trpC2 tnrA*(Cm^r^)	61
OAM843	*trpC2 amyE*::P*ilvB*-*lacZ*(−248/+26, Cm^r^::Tc^r^) *codY*(Cm^r^)	This study
OAM844	*trpC2 amyE*::P*ilvB*-*lacZ*(−248/+26, Cm^r^::Tc^r^) *tnrA*(Cm^r^)	This study
OAM845	*trpC2 amyE*::P*ilvB*-*lacZ*(−248/+26, Cm^r^::Tc^r^) *codY*(Cm^r^) *ylxR*(Km^r^)	This study
OAM846	*trpC2 amyE*::P*ilvB*-*lacZ*(−248/+26, Cm^r^::Tc^r^) *tnrA*(Cm^r^) *ylxR*(Km^r^)	This study
YXBBd	*trpC2 yxbB*::pMutin-yxbB(Em^r^)	60 (BSORF)
OAM849	*trpC2 yxbB*::pMutin-yxbB(Em^r^) *ylxR*(Km^r^)	This study
OAM847	*trpC2 yxbB*::pMutin-yxbB(Em^r^) *codY*(Cm^r^)	This study
OAM848	*trpC2 yxbB*::pMutin-yxbB(Em^r^) *codY*(Cm^r^) *ylxR*(Km^r^)	This study

Plasmids		
pX	Amp^r^ *amyE*::*xylR*-Pxyl Cm^r^	56
pX-ylxR	Amp^r^ *amyE*::*xylR*-Pxyl-*ylxR* (*ylxR* ORF with its SD) Cm^r^	This study
pET28b(+)	Km^r^ *lacI,* T7 promoter	Novagen, Inc.
pDG1729	Amp^r^ Em^r^ *thrC*::*lacZ*(Sp^r^)	57
pDG1729-ylxS	Amp^r^ Em^r^ *thrC*::P*ylxS*-*lacZ*(Sp^r^)	This study
pSG1194	Amp^r^ dsRed(Cm^r^)	59
pSG1194-ylxR	Amp^r^ *ylxR*::*gfp*(Cm^r^)	This study
pLacZ::Tc	Amp^r^ *lacZ*::Tc^r^	62
pBEST304	Tc^r^	55
pTYB11	Amp^r^ intein	New England Biolabs
pTYB11-ylxR	Amp^r^ intein-*ylxR*	This study
pIS284	Amp^r^ insertion vector to *amyE*, Cm^r^ *lacZ*	54
pIS-trmK	pIS284 carrying a promoter region of *trmK*	This study
ECE75	Amp^r^ Cm^r^::Tc^r^	Bacillus Genetic Stock Center

a ORF, open reading frame; SD, Shine-Dalgarno sequence. The numbers −284/+77 and −284/+26 indicate the nucleotide positions relative to the transcription start point.

10.1128/mSphere.00501-18.7 TABLE S3 Strains and plasmids used in the supplemental material. Download Table S3, PDF file, 0.1 MB. Copyright © 2018 Ogura and Kanesaki. 2018 Ogura and Kanesaki This content is distributed under the terms of the Creative Commons Attribution 4.0 International license.

10.1128/mSphere.00501-18.8 TABLE S4 Oligonucleotides used in this study. Download Table S4, PDF file, 0.1 MB. Copyright © 2018 Ogura and Kanesaki. 2018 Ogura and Kanesaki This content is distributed under the terms of the Creative Commons Attribution 4.0 International license.

### Strain construction.

To construct a strain carrying the *amyE*::P*ylxS*-*gfp* fusion (OAM818), first the *gfp-amyE*[front] unit was PCR amplified from strain OAM-N41 carrying *amyE*::P*tapA*-*gfp* using the oligonucleotides gfp(SD)-F/amyE-FF (53). Second, the *amyE*[back]-Cm^r^-P*ylxS* unit was PCR amplified using the oligonucleotides amyE-RR/PylxR-(SD)-gfp-R from a ligated reaction mixture of a PCR product amplified from chromosomal DNA using the oligonucleotides ylxR-Eco/ylxR-Hin treated with EcoRI/HindIII and pIS284 treated with the same enzymes (54). These fragments were combined in a final PCR using the oligonucleotides amyE-FF/amyE-RR. Final PCR products were transformed into B. subtilis 168. The *ylxQ*::Tc^r^ unit in OAM840 was constructed using PCR. Briefly Tc^r^ from pBEST304 (55) and the upstream and downstream regions of *ylxQ* with overlapping regions to Tc^r^ were amplified using primers listed in Table S4 and then combined by PCR. The unit was transformed into B. subtilis 168. Total DNA was taken from the resultant Tc^r^ strain for PCR-based confirmation of the expected chromosomal structure.

### Plasmid construction.

The plasmids used in this study are listed in Table 1 and Table S3. For PCR, chromosomal DNA was used as the template. To construct pX-ylxR, the PCR product was amplified using the oligonucleotides pX-ylxR-Spe/pX-ylxR-Bam, digested by SpeI/BamHI, and cloned into pX treated with SpeI/BamHI (56). To construct pDG1729-ylxS, the PCR product amplified by using the oligonucleotides ylxR-Eco/ylxR-Hin was digested with EcoRI/HindIII and cloned into pDG1729 treated with the same enzymes (57). To construct pTYB11-ylxR, PCR product amplified by using the oligonucleotides ylxR-chitin-F (Sap)/ylxR-chitin-R (Xh) was digested with SapI/XhoI and cloned into pTYB11 treated with the same enzymes (New England Biolabs, Ipswich, MA). To construct pSG1194-ylxR, *gfp* amplified from pMF20 using the oligonucleotides gfp-F/gfp-Xba-R and the initial PCR product with genomic regions overlapping the *gfp* gene amplified using ylxR-gfp-F/ylxR-gfp-R were combined via a second PCR using ylxR-gfp-F/gfp-Xba-R (58). The final PCR product was digested with XbaI and BamHI and cloned into the large fragment of plasmid pSG1194 lacking the Discosoma sp. red fluorescent protein DsRed, which was obtained by treatment with the same restriction enzymes, thereby generating the plasmid of interest (59).

### Purification of YlxR.

E. coli strain ER2566 bearing pTYB11-ylxR was grown in 600 ml of LB medium (100 μg/ml ampicillin) at 30°C. At an optical density at 600 nm (OD_600_) of  ≈0.8, 0.2 mM IPTG was added, and cells were further incubated for 20 h at 20°C. Chitin-binding domain- and intein-fused YlxR was purified by using chitin-coupled resin and then autoactivating the intein with dithiothreitol (DTT) according to the manufacturer’s recommendations (New England Biolabs). After SDS-PAGE analysis of the fractions, the purified protein was dialyzed against buffer containing 10 mM Tris-HCl (pH 8.0), 100 mM KCl, 10 mM MgCl_2_, 1 mM DTT, and 10% glycerol. Aliquots of purified protein were stored at −80°C.

### Electrophoretic mobility shift assay.

Purified YlxR was added to the same buffer used for dialysis containing the DNA probe in a final volume of 12 μl. Immediately after adding the protein, the reaction mixture with 2 μl of loading buffer (40% glycerol, 1× Tris-acetate-EDTA, and 2 mg/ml bromophenol blue) was applied to a 1% agarose gel, and electrophoresis was performed in Tris-acetate-EDTA buffer. DNA was detected with UV light.

### Microscopic observations.

Cells were grown in SM with or without 2% glucose, 100 μl of the culture was centrifuged, and 80 μl of the supernatant was removed. The cells were then resuspended in the remaining 20 μl. Portions (2 μl) of each sample were mounted on glass slides treated with 0.1% (wt/vol) poly-l-lysine (Sigma). If necessary, 4′,6-diamidino-2-phenylindole (DAPI) solution (1 mg/ml in water) was added to the cell suspension at 1 μg/ml. Microscopy was performed with an Olympus BX51 phase-contrast and fluorescence microscope with a 100× Plan-N objective (Olympus, Tokyo, Japan). Images were captured using a CoolSNAP HQ charge-coupled device camera (Nippon Roper, Tokyo, Japan) and Metavue 4.6r8 software (Universal Imaging, PA).

### RNA isolation and RNA-Seq analysis.

B. subtilis wild-type (OAM829) and *ylxR* disruptant (OAM830) strains were grown in 50 ml of SM with 2% glucose, and 4 ml of cell culture was sampled at T2 (i.e., a growth time of 2 h relative to the end of vegetative growth [T0]). RNA was isolated from the cells collected by centrifugation using an RNeasy minikit (Qiagen, Germantown, MD) with DNase I (TaKaRa, Shiga, Japan) treatment according to the manufacturer’s instructions. RNA-Seq was carried out by Novogene, Inc. (Hong Kong, PRC). One hundred fifty cycles of paired-end sequencing were carried out. After the sequencing reactions, the Illumina package bcl2fastq was used to process the raw data. The RNA-Seq reads were trimmed using CLC Genomics Workbench version 10.0.1 (see Table S5 in the supplemental material). The expression level of each gene was calculated by counting the mapped reads of each gene and normalized by calculating reads per kilobase per million mapped read values. A *P* value of <0.05 was considered statistically significant.

10.1128/mSphere.00501-18.9 TABLE S5 Numbers of reads obtained by next-generation sequencing. Download Table S5, PDF file, 0.1 MB. Copyright © 2018 Ogura and Kanesaki. 2018 Ogura and Kanesaki This content is distributed under the terms of the Creative Commons Attribution 4.0 International license.

### β-Galactosidase analysis.

Growth conditions and methods of β-galactosidase analysis were described previously (7, 51).

### Availability of data.

Original sequence reads were deposited in the DRA/SRA database under accession no. DRR139003 to DRR139004. Annotated data for all the genes are available at http://www.scc.u-tokai.ac.jp/iord/ogura/Ogura_and_Kanesaki2018_tableS0.xlsx.
