# Supplementary figures and images for: Newly Identified Nucleoid-Associated-Like Protein YlxR Regulates Metabolic Gene Expression in Bacillus subtilis
Source: mSphere. 2018 Oct 24;3(5):e00501-18. doi: 10.1128/mSphere.00501-18 (PMC6200986; doi:10.1128/mSphere.00501-18)

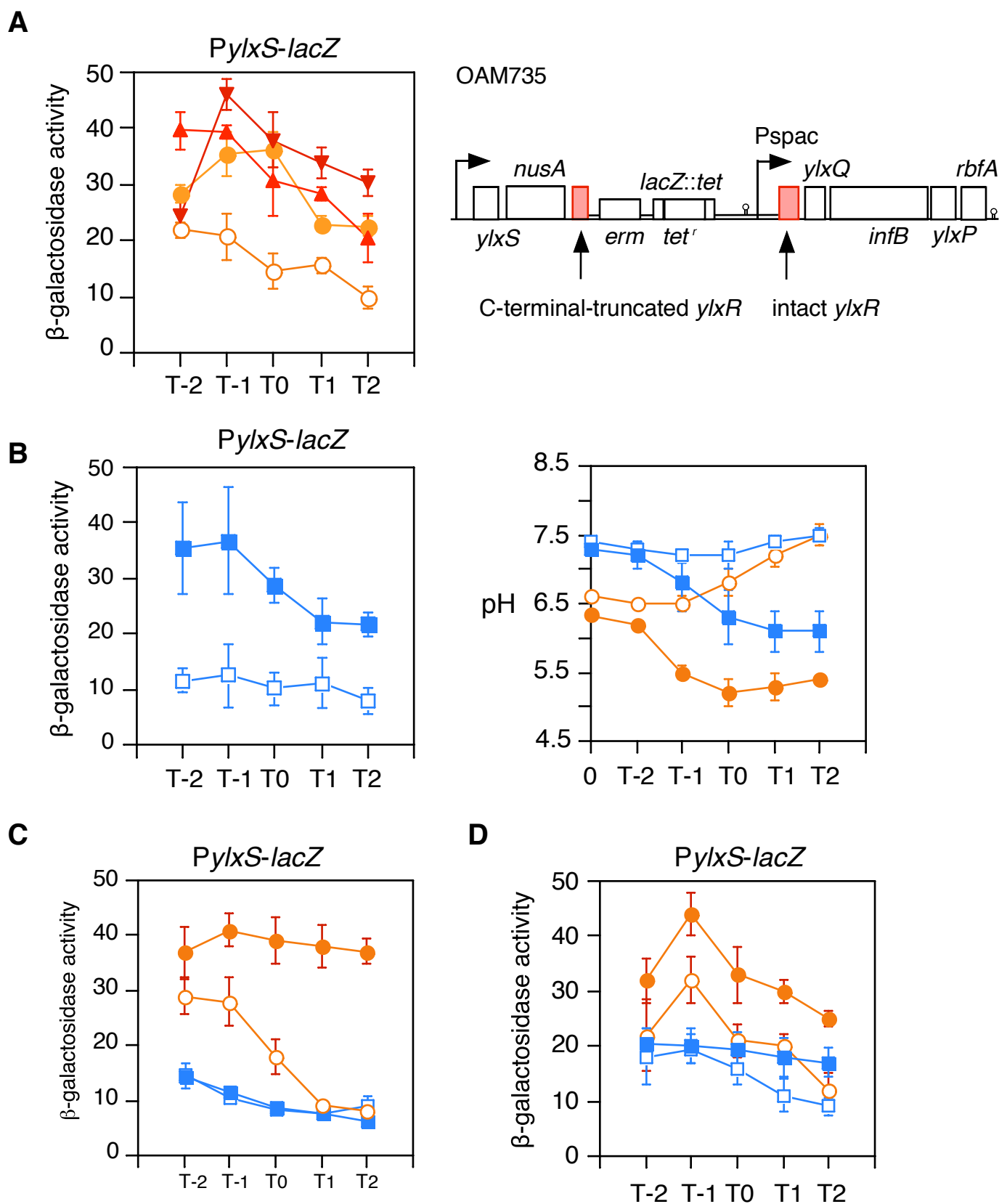

Fig. S1

Supplement: FIG S1 [file sph005182669sf1.pdf]

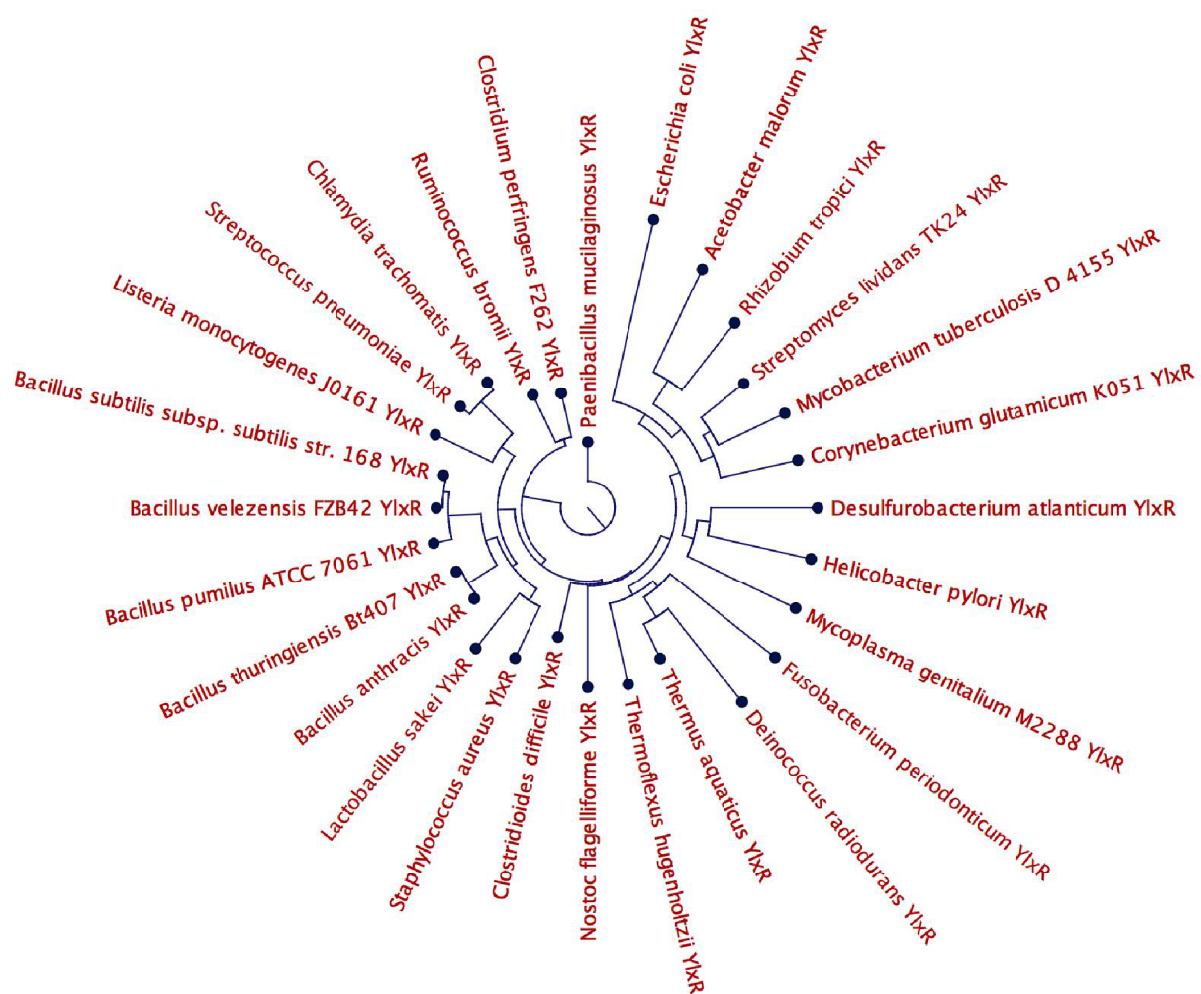

Fig. S2

Supplement: FIG S2 [file sph005182669sf2.pdf]

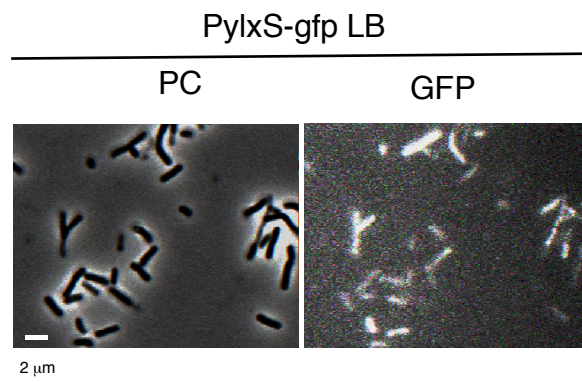

Fig. S3

Supplement: FIG S3 [file sph005182669sf3.pdf]
