# Supplementary material for: Newly Identified Nucleoid-Associated-Like Protein YlxR Regulates Metabolic Gene Expression in Bacillus subtilis
Source: mSphere. 2018 Oct 24;3(5):e00501-18. doi: 10.1128/mSphere.00501-18 (PMC6200986; doi:10.1128/mSphere.00501-18)
Supplement: TABLE S1 [file sph005182669st1.pdf]

Table S1. List of differentially expressed genes in *ylxR* .

*RPKM; Reads per kilobase per million mapped reads. *ylxR* /wild.

The CodY-, TnrA, and CcpA-regulons are from Subtiwiki.

Locus_tag	Region	Name	Averages of RPKM* Ratio	P-value	Product	Category	CodY regulon	TnrA regulon	CcpA regulon
BSU11220	1198099..1199256	*argD*	159.33	2.E-11	N-acetylornithine aminotransferase	Amino acid/ nitrogen metabolism			
BSU11240	1200381..1203473	*carB*	156.12	1.E-12	arginine-specific carbamoyl-phosphate synthetase (large subunit)	Amino acid/ nitrogen metabolism			
BSU11200	1196091..1197311	*argJ*	144.22	3.E-12	ornithine acetyltransferase; amino-acid acetyltransferase	Amino acid/ nitrogen metabolism			
BSU11210	1197326..1198102	*argB*	121.12	8.E-11	N-acetylglutamate 5-phosphotransferase (acetylglutamate kinase)	Amino acid/ nitrogen metabolism			
BSU11230	1199327..1200388	*carA*	112.05	1.E-10	arginine-specific carbamoyl-phosphate synthetase (small subunit)	Amino acid/ nitrogen metabolism			
BSU11250	1203461..1204420	*argF*	105.37	2.E-12	ornithine carbamoyltransferase	Amino acid/ nitrogen metabolism			
BSU29440	complement(3011751..3013136)	*argH*	98.98	8.E-12	argininosuccinate lyase	Amino acid/ nitrogen metabolism			
BSU29450	complement(3013133..3014344)	*argG*	85.68	6.E-18	argininosuccinate synthase	Amino acid/ nitrogen metabolism			
BSU11190	1195034..1196071	*argC*	74.14	7.E-18	N-acetylglutamate gamma-semialdehyde dehydrogenase	Amino acid/ nitrogen metabolism			
BSU32580	complement(3347919..3348821)	*frlM*	57.80	2.E-17	fructose-amino acid permease	Transporters			
BSU32590	complement(3348825..3349703)	*frlN*	52.16	7.E-19	fructose-amino acid permease	Transporters			
BSU32600	complement(3349761..3351029)	*frlO*	47.57	5.E-19	fructose amino acid-binding lipoprotein	Transporters			
BSU40780	complement(4189091..4189153)	*tetL*	45.20	3.E-05	tetracycline resistance leader peptide	Coping with stress			
BSU32570	complement(3347051..3347905)	*frlD*	31.92	9.E-19	fructoselysine kinase	Carbon metabolism			
BSU29430	complement(3011555..3011689)	*ytzD*	22.63	2.E-04	hypothetical protein	Proteins of unknown function			
BSU10230	complement(1098120..1098260)	*yhfH*	21.53	3.E-03	hypothetical protein	Proteins of unknown function			
BSU32610	complement(3351110..3352096)	*frlB*	20.87	9.E-21	fructoselysine-6-P-deglycase	Carbon metabolism			
BSU23980	complement(2492029..2492796)	*artP*	19.13	4.E-32	High affinity arginine ABC transporter binding lipoprotein	Transporters			
BSU23970	complement(2491289..2491948)	*artQ*	18.15	8.E-13	High affinity arginine ABC transporter (permease)	Transporters			
BSU23960	complement(2490574..2491296)	*artR*	17.71	2.E-10	High affinity arginine ABC transporter (ATP-binding protein)	Transporters			
BSU20150	complement(2168114..2168461)	*yosE*	17.14	2.E-02	hypothetical protein; phage SPbeta	Prophages			
BSU09700	1045037..1045198	*yheJ*	15.99	3.E-01	hypothetical protein	Regulation of gene expression			
BSU24400	complement(2535544..2535945)	*spoIIIAD*	15.94	4.E-02	stage III sporulation protein	Protein synthesis, modification and degradation			
BSU32550	complement(3345013..3346116)	*yurJ*	15.09	3.E-14	putative multiple sugar ABC transporter (ATP-binding protein)	Transporters			
BSU12519	1321848..1322027	*yjzJ*	13.93	2.E-02	conserved hypothetical protein	Prophages			
BSU11928	1265530..1265661	*yjzF*	12.18	4.E-02	hypothetical protein	Proteins of unknown function			
BSU19639	2136373..2136498	*yoyE*	11.75	3.E-02	conserved hypothetical protein	Proteins of unknown function			
BSU04880	536096..536365	*ydcS*	9.35	3.E-02	conserved hypothetical protein; mobile element region	Mobile genetic elements			
BSU18220	complement(1951228..1952010)	*yngF*	9.35	3.E-02	putative Methylglutaconyl-CoA hydratase	Amino acid/ nitrogen metabolism			
BSU33820	complement(3469184..3469837)	*opuCB*	9.26	1.E-07	glycine betaine/carnitine/choline/choline sulfate ABC transporter (permease)	Transporters			
BSU40180	complement(4127498..4127647)	*yydF*	9.12	4.E-10	peptide controlling LiaRS	Regulation of gene expression			
BSU01920	214175..215407	*skfB*	8.85	1.E-16	synthesis of sporulation killing factor A	Coping with stress			
BSU33810	complement(3468253..3469164)	*opuCC*	8.56	1.E-07	glycine betaine/carnitine/choline/choline sulfate ABC transporter (osmoprotectant-binding lipoprotein)	Transporters			
BSU01910	213941..214108	*skfA*	8.50	5.E-09	sporulation killing factor A	Coping with stress			
BSU12940	1362174..1363136	*dppC*	8.36	2.E-05	dipeptide ABC transporter (permease)	Transporters			
BSU31321	3218525..3218857	*mstX*	8.35	2.E-02	atypical membrane-integrating protein (Mistic protein)	Protein synthesis, modification and degradation			
BSU33830	complement(3469860..3471002)	*opuCA*	8.23	9.E-08	glycine betaine/carnitine/choline/choline sulfate ABC transporter (ATP-binding protein)	Transporters			
BSU33800	complement(3467546..3468235)	*opuCD*	8.10	1.E-08	glycine betaine/carnitine/choline/choline sulfate ABC transporter (permease)	Transporters			
BSU40770	complement(4187681..4189057)	*tetB*	7.93	1.E-24	multifunctional tetracycline-metal/H+ antiporter and Na+(K+)/H+ antiporter	Transporters			
BSU32560	3346298..3347026	*frlR*	7.66	1.E-09	FrlR transcriptional regulator (GntR family)	Carbon metabolism			
BSU01935	215404..216894	*skfC*	6.93	2.E-12	sporulation killing factor biosynthesis and export	Coping with stress			
BSU01990	220279..221256	*ybdG*	6.30	3.E-09	putative hydrolase/transferase	Membrane proteins			
BSU12950	1363141..1364148	*dppD*	6.14	4.E-07	dipeptide ABC transporter (ATP-binding protein)	Transporters			
BSU05560	complement(602185..602427)	*ydgA*	6.10	1.E-03	conserved hypothetical protein	Sporulation			
BSU01960	217697..219040	*skfF*	5.30	3.E-11	sporulation killing factor biosynthesis and export; ABC transporter (permease)	Transporters			
BSU01980	219607..220032	*skfH*	5.11	2.E-11	sibling killing effect; sporulation killing factor biosynthesis and export	Prophages			
BSU39920	4098926..4101169	*asnH*	5.02	4.E-11	asparagine synthetase (glutamine-hydrolyzing)	Amino acid/ nitrogen metabolism			
BSU12960	1364151..1365800	*dppE*	4.97	4.E-06	dipeptide ABC transporter (dipeptide-binding lipoprotein)	Transporters			
BSU37760	complement(3875780..3877192)	*rocC*	4.94	9.E-03	arginine/ornithine permease	Transporters			
BSU39910	4098423..4098905	*yxnB*	4.91	3.E-10	hypothetical protein	Proteins of unknown function			
BSU19730	complement(2143660..2144349)	*yodS*	4.89	1.E-02	putative aminoacyloate CoA-transferase	Sporulation			
BSU39930	4101166..4102365	*yxaM*	4.89	4.E-10	putative efflux transporter	Coping with stress			

BSU19749	complement(2145820..2145933)	yoyG	4.88	2.E-02	putative sporulation protein	Proteins of unknown function			
BSU24140	complement(2509654..2510772)	mmgD	4.87	1.E-01	2-methylcitrate synthase/citrate synthase III	Amino acid/ nitrogen metabolism			
BSU33710	complement(3460503..3461423)	opuBC	4.72	5.E-03	choline ABC transporter (choline-binding lipoprotein)	Transporters			
BSU15960	complement(1671166..1671651)	ylqB	4.64	2.E-10	conserved hypothetical protein	Regulation of gene expression			
BSU01950	216913..217632	skfE	4.61	4.E-10	sporulation killing factor biosynthesis and export; ABC transporter (binding protein)	Transporters			
BSU12930	1361242..1362168	dppB	4.52	3.E-04	dipeptide ABC transporter (permease)	Transporters			
BSU25060	2588701..2589000	yqfZ	4.51	2.E-03	factor involved in motility	Sporulation			
BSU01970	219087..219602	skfG	4.51	4.E-11	sporulation killing factor biosynthesis and export	Prophages			
BSU06038	complement(652713..653018)	ydzT_5	4.35	1.E-04	hypothetical protein; putative integrase (fragment); N-terminal part of YdzT	Prophages			
BSU40573	complement(4171396..4171635)	yyzJ	4.34	8.E-03	hypothetical protein	Proteins of unknown function			
BSU39890	4097416..4098150	yxbB	4.18	4.E-10	putative S-adenosylmethionine-dependent methyltransferase	Proteins of unknown function			
BSU24090	complement(2503765..2504664)	ptb	4.17	7.E-05	phosphate butyryl coenzyme A transferase	Amino acid/ nitrogen metabolism			
BSU37770	complement(3877192..3878892)	rocB	4.16	3.E-02	putative N-deacylase involved in arginine and ornithine utilization	Amino acid/ nitrogen metabolism			
BSU38080	complement(3907012..3907314)	ywcI	4.03	9.E-05	hypothetical protein	Proteins of unknown function			
BSU10740	complement(1151166..1152089)	yisJ	3.92	6.E-03	putative spore coat protein	Sporulation			
BSU33770	3465776..3466387	spbC	3.89	1.E-09	killing factor SdpC	Coping with stress			
BSU30290	3100031..3100861	amyC	3.88	4.E-03	maltose and multiple sugars ABC transporter (permease)	Transporters			
BSU20300	complement(2174104..2174319)	yorP	3.88	4.E-03	hypothetical protein; phage SPbeta	Prophages			
BSU07480	820867..822330	yfmG	3.88	7.E-07	conserved hypothetical protein	Phosphoproteins			
BSU39900	4098150..4098419	yxbA	3.81	1.E-08	conserved hypothetical protein	Poorly characterized/ putative enzymes			
BSU40640	complement(4177756..4178145)	yybH	3.80	3.E-05	conserved hypothetical protein	Proteins of unknown function			
BSU19850	complement(2153171..2153356)	yotK	3.79	3.E-02	conserved hypothetical protein; phage SPbeta	Prophages			
BSU30970	complement(3168624..3169766)	glgC	3.77	6.E-05	glucose-1-phosphate adenylyltransferase (ADP-glucose pyrophosphorylase) subunit alpha	Additional metabolic pathways			
BSU15310	1603779..1604708	spoIIGA	3.72	5.E-05	protease processing pro-sigma-E	Protein synthesis, modification and degradation			
BSU00640	70538..73021	spoIIE	3.65	3.E-04	SpoIIAA-phosphate serine phosphatase	Protein synthesis, modification and degradation			
BSU40260	complement(4137362..4137610)	yycQ	3.61	4.E-04	putative conserved membrane protein	Sporulation			
BSU07780	complement(847498..848652)	yfkR	3.57	4.E-04	putative spore germination protein	Sporulation			
BSU_rRNA_30	635433..636987	rrnE-16S	3.55	8.E-03	ribosomal RNA-16S	Protein synthesis, modification and degradation			
BSU20000	2158439..2158684	yosU	3.53	4.E-02	hypothetical protein	Prophages			
BSU31570	3243657..3244616	nupQ	3.51	4.E-04	permease of ABC guanosine transporter	Transporters			
BSU13000	1368844..1369833	ykfD	3.51	1.E-03	putative cell wall oligopeptide ABC transporter (ATP binding protein)	Transporters			
BSU24080	complement(2502659..2503753)	bcd	3.49	1.E-03	branched-chain amino acid dehydrogenase	Amino acid/ nitrogen metabolism			
BSU12980	1366844..1367944	ykfB	3.47	6.E-04	L-Ala-D/L-Glu epimerase	Cell envelope and cell division			
BSU30950	complement(3166118..3167572)	glgA	3.45	2.E-06	bacterial glycogen (starch) synthase	Additional metabolic pathways			
BSU33410	3427802..3428287	yvgO	3.44	2.E-02	conserved hypothetical protein	Coping with stress			
BSU40170	complement(4126481..4127440)	yydG	3.43	3.E-04	putative AdoMet radical enzyme	Protein synthesis, modification and degradation			
BSU28060	complement(2863294..2864292)	spoIIB	3.42	4.E-08	spatial and temporal regulator of the dissolution of septal peptidoglycan during engulfment (stage II sporulation)	Sporulation			
BSU03900	441571..442881	gabT	3.41	2.E-04	4-aminobutyrate aminotransferase	Amino acid/ nitrogen metabolism			
BSU06037	complement(652511..652720)	ydzT_4	3.39	5.E-05	hypothetical protein; putative integrase (fragment); internal part of YdzT	Prophages			
BSU26100	complement(2681627..2682130)	yqbI	3.38	2.E-02	conserved hypothetical protein; skin element	Prophages			
BSU15320	1604771..1605490	sigE	3.37	2.E-05	RNA polymerase sporulation-specific sigma-29 factor (sigma-E)	RNA synthesis and degradation			
BSU31550	3241085..3242617	nupO	3.35	1.E-03	guanosine ABC transporter (ATP-binding protein)	Transporters			
BSU09710	1045318..1047075	yheI	3.34	2.E-10	ABC transporter (ATP-binding protein) involved in the signalling pathway that activates KinA during sporulation initiation	Transporters			
BSU_misc_RNA_86	4096997..4097409	srlX	3.33	3.E-09					
BSU02050	225064..226248	ybdO	3.24	1.E-05	putative phage protein	Proteins of unknown function			
BSU16610	1733410..1733685	ylxR	3.23	1.E-08	putative RNA binding protein; putative new fold	Proteins of unknown function			
BSU11260	1204506..1204685	yjzC	3.22	5.E-04	conserved hypothetical protein	Proteins of unknown function			
BSU21330	2247889..2248335	yomK	3.22	1.E-04	hypothetical protein; phage SPbeta	Prophages			
BSU20310	complement(2174352..2174549)	yorO	3.20	1.E-02	hypothetical protein; phage SPbeta	Prophages			
BSU31250	complement(3208280..3210268)	tlpA	3.17	1.E-05	methyl-accepting chemotaxis protein	Exponential and early post-exponential lifestyles			
BSU31560	3242610..3243656	nupP	3.13	9.E-04	permease of ABC guanosine transporter	Transporters			
BSU26559	complement(2714590..2714805)	yrzN	3.12	7.E-03	hypothetical protein	Proteins of unknown function			
BSU30960	complement(3167569..3168600)	glgD	3.12	2.E-05	glucose-1-phosphate adenylyltransferase (ADP-glucose pyrophosphorylase) beta subunit	Additional metabolic pathways			
BSU40160	complement(4125742..4126500)	yydH	3.12	6.E-05	putative membrane metalloprotease	Protein synthesis, modification and degradation			
BSU12450	complement(1317157..1317414)	yjpA	3.11	5.E-04	putative enzyme	Proteins of unknown function			
BSU33720	complement(3461435..3462088)	opuBB	3.09	3.E-02	choline ABC transporter (permease)	Transporters			

BSU39860	4093980..4095317	*aldX*	3.08	1.E-04	putative aldehyde dehydrogenase	Poorly characterized/ putative enzymes			
BSU39720	complement(4078173..4079066)	*iolE*	3.08	9.E-02	2-keto-myo-inositol dehydratase	Carbon metabolism			
BSU30980	complement(3169763..3171646)	*glgB*	3.08	9.E-05	1,4-alpha-glucan branching enzyme	Additional metabolic pathways			
BSU_misc_RNA_32	complement(2095909..2096111)	*ssrSA*	3.05	3.E-05		Regulation of gene expression			
BSU28890	2953795..2954460	*yscB*	3.04	1.E-05	putative lipoprotein	Proteins of unknown function			
BSU12990	1367941..1368831	*ykfC*	3.04	2.E-03	gamma-D-glutamyl-L-diaminoacid endopeptidase	Cell envelope and cell division			
BSU09720	1047072..1049093	*yheH*	3.03	2.E-09	ABC transporter (ATP-binding protein) involved in the signalling pathway that activates KinA during sporulation initiation	Transporters			
BSU33730	complement(3462105..3463250)	*opuBA*	3.02	1.E-03	choline ABC transporter (ATP-binding protein)	Transporters			
BSU36420	complement(3748421..3748702)	*spoIIID*	3.01	1.E-02	transcriptional regulator	Regulation of gene expression			
BSU12330	1304442..1305461	*yjmD*	2.94	2.E-01	putative oxidoreductase	Carbon metabolism			
BSU38400	3939869..3941806	*epr*	2.93	6.E-05	extracellular serine protease	Amino acid/ nitrogen metabolism			
BSU23450	complement(2443429..2444196)	*sigF*	2.92	9.E-06	RNA polymerase sporulation-specific sigma factor (sigma-F)	RNA synthesis and degradation			
BSU02000	221258..221929	*ybdJ*	2.90	1.E-05	two-component response regulator [YbdK]	Regulation of gene expression			
BSU02010	221950..222912	*ybdK*	2.88	7.E-06	two-component sensor histidine kinase [YbdJ]	Protein synthesis, modification and degradation			
BSU12970	1365888..1366847	*ldcA*	2.86	1.E-03	muropeptide L,D-carboxypeptidase	Coping with stress			
BSU03220	347150..348571	*putP*	2.86	2.E-03	proline permease	Transporters			
BSU11390	1215243..1216193	*appB*	2.86	7.E-05	oligopeptide ABC transporter (permease)	Transporters			
BSU40660	complement(4179163..4180377)	*yybF*	2.85	4.E-03	putative permease	Coping with stress			
BSU37750	complement(3874332..3875570)	*ywfA*	2.85	9.E-03	putative efflux transporter	Coping with stress			
BSU39880	complement(4095915..4096907)	*yxbC*	2.83	1.E-05	conserved hypothetical protein	Proteins of unknown function			
BSU30760	complement(3144267..3145019)	*mntB*	2.80	2.E-02	manganese ABC transporter (ATP-binding protein)	Transporters			
BSU18610	complement(2029429..2031114)	*yoaH*	2.80	5.E-04	putative methyl-accepting chemotaxis protein	Exponential and early post-exponential lifestyles			
BSU11929	1265677..1265850	*yjzG*	2.80	5.E-03	hypothetical protein	Proteins of unknown function			
BSU_misc_RNA_18	complement(1385736..1385891)	*mswB*	2.79	2.E-04					
BSU22160	complement(2329515..2329706)	*yptA*	2.78	4.E-05	hypothetical protein	Sporulation			
BSU34120	complement(3501651..3502940)	*ganB*	2.77	2.E-02	secreted arabinogalactan oligomer endo-hydrolase	Carbon metabolism			
BSU33700	complement(3459806..3460486)	*opuBD*	2.76	2.E-03	choline ABC transporter (permease)	Coping with stress			
BSU11381	1213537..1214001	*appA_1*	2.74	1.E-04	oligopeptide ABC transporter (oligopeptide-binding lipoprotein); N-terminal part of AppA	Transporters			
BSU06036	complement(652290..652418)	*ydzT_3*	2.73	1.E-02	hypothetical protein; putative integrase (fragment); internal part of YdzT	Prophages			
BSU06048	654071..654343	*ydzU*	2.73	3.E-03	hypothetical protein	Prophages			
BSU_misc_RNA_2	26379..26732	*scr*	2.73	4.E-07		Protein synthesis, modification and degradation			
BSU39750	complement(4082030..4082845)	*iolB*	2.73	9.E-03	5-deoxy-D-glucuronic acid isomerase	Carbon metabolism			
BSU06073	complement(657793..658062)	*ydzW_1*	2.71	4.E-04	putative phosphoglucomutase; C-terminal part of YdzW	Prophages			
BSU32849	3372569..3372715	*yuzL*	2.71	2.E-02	conserved hypothetical protein	Proteins of unknown function			
BSU18820	2050689..2050952	*yobB*	2.68	1.E-05	putative transcriptional regulator from bacteriophage	Proteins of unknown function			
BSU40150	complement(4124963..4125592)	*yydI*	2.68	2.E-05	ABC transporter (ATP-binding protein)	Transporters			
BSU05540	601019..601726	*ydfS*	2.68	1.E-02	conserved hypothetical protein	Sporulation			
BSU12920	1360401..1361225	*dppA*	2.66	2.E-04	D-alanyl-aminopeptidase	Amino acid/ nitrogen metabolism			
BSU11400	1216210..1217121	*appC*	2.65	3.E-04	oligopeptide ABC transporter (permease)	Transporters			
BSU33750	3464289..3464765	*sdpA*	2.62	1.E-02	export of killing factor	Coping with stress			
BSU12500	complement(1320570..1321166)	*xkdA*	2.62	1.E-04	PBSX phage protein, putative peptidase	Prophages			
BSU08330	907968..909125	*yfiN*	2.61	4.E-03	putative ABC transporter (permease)	Transporters			
BSU06049	654333..654692	*ydzV*	2.61	3.E-04	conserved hypothetical protein; phage terminase (fragment)	Prophages			
BSU07735	844253..844645	*yflB*	2.61	2.E-04	conserved hypothetical protein	Proteins of unknown function			
BSU18670	complement(2037601..2038779)	*yoaN*	2.60	7.E-03	oxalate decarboxylase	Sporulation			
BSU30940	complement(3163735..3166131)	*glgP*	2.59	3.E-04	glycogen phosphorylase	Additional metabolic pathways			
BSU04070	459049..459822	*ycsI*	2.58	6.E-03	conserved hypothetical protein	Proteins of unknown function			
BSU40140	complement(4124220..4124942)	*yydJ*	2.57	4.E-05	putative permease for export of a regulatory peptide	Transporters			
BSU19579	2130177..2130377	*yoyD*	2.57	3.E-02	putative exported protein	Proteins of unknown function			
BSU13180	complement(1383320..1385608)	*metE*	2.56	1.E-04	cobalamin-independent methionine synthase	Amino acid/ nitrogen metabolism			
BSU23460	complement(2444208..2444648)	*spoIIAB*	2.56	6.E-05	anti-sigma factor (antagonist of sigma(F)) and serine kinase	Protein synthesis, modification and degradation			
BSU31260	complement(3210445..3212433)	*mcpB*	2.56	4.E-04	methyl-accepting chemotaxis protein	Exponential and early post-exponential lifestyles			
BSU17310	complement(1865512..1865787)	*ymaG*	2.55	2.E-02	inner spore coat protein; cell wall associated protein	Sporulation			
BSU11370	1212460..1213449	*appF*	2.55	2.E-05	oligopeptide ABC transporter (ATP-binding protein)	Transporters			
BSU13950	1463628..1465595	*mcpC*	2.54	9.E-04	methyl-accepting chemotaxis protein	Exponential and early post-exponential lifestyles			
BSU31240	complement(3206169..3208154)	*mcpA*	2.54	2.E-04	methyl-accepting chemotaxis protein	Exponential and early post-exponential lifestyles			
BSU23470	complement(2444645..2444998)	*spoIIAA*	2.50	3.E-04	anti-anti-sigma factor (antagonist of SpoIIAB)	Regulation of gene expression			
BSU22550	complement(2363913..2364587)	*qcrB*	2.50	2.E-04	menaquinol:cytochrome c oxidoreductase (cytochrome b subunit)	Electron transport and ATP synthesis			

BSU14910	1563437..1564060	ctaE	2.50	9.E-05	cytochrome caa3 oxidase (subunit III)	Electron transport and ATP synthesis			
BSU11382	1214001..1215167	appA_2	2.48	3.E-04	oligopeptide ABC transporter (oligopeptide-binding lipoprotein); C-terminal part of AppA	Transporters			
BSU40320	complement(4141711..4142601)	argI	2.48	4.E-02	arginase	Amino acid/ nitrogen metabolism			
BSU11900	complement(1261426..1262616)	yjcL	2.46	3.E-05	putative integral inner membrane protein; possibly aquaporin-related	Proteins of unknown function			
BSU40120	complement(4122619..4122849)	yyzE	2.45	4.E-03	putative phosphotransferase system enzyme IIA component	Transporters			
BSU08280	902506..903687	yfiI	2.45	9.E-02	putative oxidoreductase	Proteins of unknown function			
BSU26200	complement(2689594..2690313)	yqaS	2.44	4.E-04	putative phage-related terminase small subunit; skin element	Prophages			
BSU07760	complement(846182..847258)	yfkT	2.44	1.E-02	putative spore germination integral inner membrane protein	Sporulation			
BSU07750	844770..846185	yflA	2.43	2.E-03	putative aminoacid transporter	Transporters			
BSU33760	3464762..3465733	sdpB	2.43	1.E-02	exporter of killing factor SpbC	Coping with stress			
BSU07490	complement(822903..823703)	yfmF	2.42	2.E-04	iron-dicitrate ABC transporter (ATP-binding protein)	Transporters			
BSU25520	complement(2632882..2633220)	yqxA	2.41	1.E-02	conserved hypothetical protein	Sporulation			
BSU13350	complement(1398975..1400096)	ykoN	2.41	8.E-03	putative glycosyltransferase	Sporulation			
BSU25750	complement(2652387..2652797)	nucB	2.41	1.E-03	nuclease	Nucleotide metabolism			
BSU31960	complement(3280519..3287655)	dhbF	2.40	9.E-03	siderophore 2,3-dihydroxybenzoate-glycine-threonine trimeric ester bacillibactin synthetase	Homeostasis			
BSU06839	752079..752252	phrH	2.38	4.E-03	hexapeptide inhibitor of regulatory cascade	Regulation of gene expression			
BSU14900	1561569..1563437	ctaD	2.37	1.E-04	cytochrome caa3 oxidase (subunit I)	Electron transport and ATP synthesis			
BSU19400	complement(2114742..2115332)	sodC	2.37	1.E-04	superoxide dismutase (exported lipoprotein)	Coping with stress			
BSU10930	complement(1171755..1172465)	yitB	2.37	7.E-03	putative phospho-adenylylsulfate sulfotransferase	Additional metabolic pathways			
BSU10950	1173333..1174091	slpS	2.34	2.E-02	phosphosulfolactate synthase	Sporulation			
BSU13360	1400188..1400739	ykoP	2.34	1.E-02	conserved hypothetical protein	Sporulation			
BSU11839	1256109..1256363	yjzE	2.34	3.E-02	hypothetical protein	Coping with stress			
BSU11950	1267129..1267413	yjcQ	2.34	3.E-03	conserved hypothetical protein	Proteins of unknown function			
BSU26120	complement(2682486..2682881)	yqbG	2.34	4.E-04	conserved hypothetical protein; skin element	Prophages			
BSU18690	complement(2039610..2040365)	yoaP	2.34	4.E-02	conserved hypothetical protein	Proteins of unknown function			
BSU19640	complement(2136538..2136852)	yodL	2.33	5.E-06	conserved hypothetical protein	Cell envelope and cell division			
BSU14010	1473605..1474516	cheV	2.33	2.E-04	coupling protein and response regulator for CheA activity in response to attractants	Regulation of gene expression			
BSU11990	complement(1269733..1270080)	yjdB	2.32	6.E-02	putative exported protein	Proteins of unknown function			
BSU18260	complement(1956218..1957360)	yngJ	2.32	4.E-02	acyl-CoA dehydrogenase, short-chain specific	Amino acid/ nitrogen metabolism			
BSU33690	3458066..3459766	yvaQ	2.32	5.E-04	putative methyl-accepting transducer	Exponential and early post-exponential lifestyles			
BSU14930	1564422..1565315	ctaG	2.31	9.E-05	cytochrome aa(3) assembly factor	Electron transport and ATP synthesis			
BSU30620	3134144..3134956	ytlD	2.30	7.E-03	putative permease of ABC transporter	Transporters			
BSU26240	complement(2692645..2692851)	yqaO	2.30	3.E-02	conserved hypothetical protein; skin element	Prophages			
BSU18480	complement(2016845..2017738)	proH	2.28	3.E-03	pyrroline-5-carboxylate reductase	Amino acid/ nitrogen metabolism			
BSU12120	complement(1283169..1283336)	yjfB	2.27	2.E-03	conserved hypothetical protein	Proteins of unknown function			
BSU39760	complement(4082920..4084383)	mmsA	2.26	7.E-03	methylmalonate-semialdehyde dehydrogenase	Carbon metabolism			
BSU37480	3847348..3847821	ywhH	2.25	3.E-05	putative RNA-binding protein	Proteins of unknown function			
BSU38090	3907844..3910264	vpr	2.25	3.E-03	extracellular serine protease	Amino acid/ nitrogen metabolism			
BSU03210	345479..347026	putC	2.24	1.E-02	1-pyrroline-5-carboxylate dehydrogenase	Amino acid/ nitrogen metabolism			
BSU31820	complement(3264265..3264501)	yuzF	2.24	3.E-02	conserved hypothetical protein	Sporulation			
BSU30750	complement(3142954..3144261)	mntC	2.23	4.E-03	manganese ABC transporter (permease)	Transporters			
BSU33790	complement(3467054..3467326)	sdpR	2.23	2.E-03	transcriptional regulator (ArsR family)	Regulation of gene expression			
BSU22540	complement(2363111..2363878)	qcrC	2.22	1.E-03	menaquinol:cytochrome c oxidoreductase (cytochrome cc subunit)	Electron transport and ATP synthesis			
BSU31230	complement(3204067..3206055)	tlpB	2.22	4.E-03	methyl-accepting chemotaxis protein	Exponential and early post-exponential lifestyles			
BSU10730	complement(1150850..1151020)	yisI	2.22	8.E-03	Spo0A-P phosphatase	Protein synthesis, modification and degradation			
BSU22530	complement(2362407..2362964)	ypjA	2.22	2.E-04	putative integral inner membrane protein	Membrane proteins			
BSU30740	complement(3142077..3142964)	mntD	2.22	1.E-03	manganese ABC transporter (permease)	Transporters			
BSU14160	1487504..1488397	ykuO	2.22	1.E-02	conserved hypothetical protein	Proteins of unknown function			
BSU39129	complement(4018786..4018974)	yxzI	2.22	2.E-02	hypothetical protein	Proteins of unknown function			
BSU06040	653432..653812	ydiM	2.22	1.E-03	hypothetical protein	Prophages			
BSU35440	complement(3640632..3641051)	yvyF	2.20	4.E-03	putative regulator of flagella formation	Exponential and early post-exponential lifestyles			
BSU00660	73809..74825	yabT	2.19	6.E-03	putative serine/threonine-protein kinase	Protein synthesis, modification and degradation			
BSU10790	1157237..1159081	asnO	2.18	1.E-02	asparagine synthetase	Amino acid/ nitrogen metabolism			
BSU02510	274029..275561	garD	2.18	1.E-02	D-galactarate dehydratase	Carbon metabolism			
BSU16080	1679977..1680258	ylqH	2.18	6.E-04	putative flagellar biosynthesis protein	Exponential and early post-exponential lifestyles			
BSU40630	complement(4176900..4177688)	yybI	2.18	5.E-04	inner spore coat protein	Sporulation			
BSU14170	1488413..1488868	ykuP	2.18	6.E-03	short-chain flavodoxin	Electron transport and ATP synthesis			
BSU39870	complement(4095356..4095835)	yxbD	2.18	5.E-05	putative acetyltransferase	Proteins of unknown function			

BSU08310	905816..906751	*yfiL*	2.17	3.E-01	putative ABC transporter (ATP-binding protein)	Transporters			
BSU06074	complement(658061..658189)	*ydzW_2*	2.17	1.E-02	putative phosphoglucomutase; internal part of YdzW	Prophages			
BSU29500	complement(3018309..3018764)	*ytfJ*	2.17	1.E-02	conserved hypothetical protein	Sporulation			
BSU26210	complement(2690381..2690845)	*yqaR*	2.17	7.E-03	hypothetical protein; skin element	Prophages			
BSU30890	complement(3159258..3159689)	*ytxO*	2.17	2.E-02	outer spore coat protein	Sporulation			
BSU28270	complement(2891020..2892117)	*leuB*	2.16	3.E-05	3-isopropylmalate dehydrogenase	Amino acid/ nitrogen metabolism			
BSU19750	complement(2146013..2146792)	*cgeE*	2.15	2.E-03	protein involved in maturation of the outermost layer of the spore	Sporulation			
BSU10670	complement(1148494..1148712)	*gerPF*	2.15	4.E-02	spore germination protein	Sporulation			
BSU19580	2130377..2131867	*yodF*	2.15	4.E-04	putative Na+/metabolite permease	Transporters			
BSU22560	complement(2364589..2365092)	*qcrA*	2.14	2.E-04	menaquinol:cytochrome c oxidoreductase (iron-sulfur subunit)	Electron transport and ATP synthesis			
BSU18470	complement(2015733..2016848)	*proJ*	2.13	4.E-03	glutamate 5-kinase	Amino acid/ nitrogen metabolism			
BSU24070	complement(2501549..2502640)	*buk*	2.12	1.E-02	branched-chain fatty-acid kinase	Amino acid/ nitrogen metabolism			
BSU38850	3989331..3989873	*yxkC*	2.11	2.E-02	conserved hypothetical protein	Proteins of unknown function			
BSU10920	complement(1170473..1171642)	*yitA*	2.10	2.E-02	putative sulfate adenylyltransferase	Additional metabolic pathways			
BSU07460	complement(819311..820531)	*yfmI*	2.10	3.E-04	putative efflux transporter	Transporters			
BSU39980	complement(4106245..4107258)	*qodI*	2.10	3.E-03	quercetin dioxygenase	Coping with stress			
BSU32930	complement(3378800..3379087)	*yusU*	2.09	1.E-03	conserved hypothetical protein	Proteins of unknown function			
BSU26190	complement(2688306..2689601)	*yqaT*	2.09	8.E-03	putative phage-related terminase large subunit; skin element	Prophages			
BSU31540	3239930..3241009	*nupN*	2.08	3.E-02	lipoprotein involved in guanosine transport	Nucleotide metabolism			
BSU37510	3849818..3851893	*pbpG*	2.08	3.E-02	sporulation specific penicillin-binding protein	Cell envelope and cell division			
BSU11360	1211477..1212463	*appD*	2.07	4.E-04	oligopeptide ABC transporter (ATP-binding protein)	Transporters			
BSU03200	344551..345462	*putB*	2.07	2.E-02	proline oxidase	Amino acid/ nitrogen metabolism			
BSU32540	3344113..3344979	*bsn*	2.07	3.E-04	extracellular ribonuclease	Additional metabolic pathways			
BSU19650	complement(2136913..2137524)	*yodM*	2.06	1.E-02	putative phospholipid phosphatase	Proteins of unknown function			
BSU22170	complement(2330075..2331232)	*ypsC*	2.06	1.E-05	putative methylase with RNA interaction domain	Protein synthesis, modification and degradation			
BSU20950	complement(2214972..2215199)	*yopB*	2.06	3.E-02	putative transcriptional regulator; phage SPbeta	Prophages			
BSU10410	complement(1116583..1116816)	*yhzC*	2.05	6.E-03	hypothetical protein	Proteins of unknown function			
BSU25840	2660330..2660464	*phrE*	2.05	4.E-05	regulator of the activity of phosphatase RapE	Regulation of gene expression			
BSU11410	1217326..1218078	*yjbA*	2.05	6.E-04	putative nucleic acid binding protein	Sporulation			
BSU19180	2089396..2090454	*des*	2.04	7.E-04	fatty acid desaturase	Lipid metabolism			
BSU12050	complement(1277062..1277457)	*yjdH*	2.04	1.E-02	hypothetical protein	Sporulation			
BSU08490	complement(924210..924401)	*yfhD*	2.03	2.E-02	conserved hypothetical protein	Coping with stress			
BSU10380	complement(1112620..1113918)	*hemAT*	2.02	8.E-04	haem-based dioxygen sensor	Exponential and early post-exponential lifestyles			
BSU28320	2897788..2898123	*ysnD*	2.02	3.E-02	inner spore coat protein	Sporulation			
BSU06260	679827..680873	*ydjN*	2.02	6.E-03	putative membrane protein	Proteins of unknown function			
BSU13940	complement(1462813..1463493)	*ykwB*	2.02	8.E-05	putative acetyltransferase	Sporulation			
BSU12110	complement(1282571..1283044)	*yjfA*	2.01	4.E-03	conserved hypothetical protein	Sporulation			
BSU28280	complement(2892138..2893694)	*leuA*	2.01	2.E-04	2-isopropylmalate synthase	Amino acid/ nitrogen metabolism			
BSU16070	1678250..1679980	*ylqG*	2.01	1.E-04	putative glycosyltransferase	Proteins of unknown function			
BSU35630	complement(3660648..3662765)	*lytB*	2.01	1.E-03	modifier protein of major autolysin LytC	Cell envelope and cell division			
BSU06300	complement(683462..685003)	*cotA*	2.00	2.E-02	outer spore coat copper-dependent laccase	Sporulation			
BSU34930	complement(3588185..3589360)	*hisZ*	0.04	1.E-20	histidyl-tRNA synthetase-like component of ATP phophoribosyltransferase	Protein synthesis, modification and degradation			
BSU34920	complement(3587551..3588192)	*hisG*	0.07	3.E-09	ATP phosphoribosyltransferase	Amino acid/ nitrogen metabolism			
BSU34910	complement(3586271..3587554)	*hisD*	0.10	4.E-05	histidinol dehydrogenase	Amino acid/ nitrogen metabolism			
BSU08880	965909..966178	*rpsNB*	0.10	1.E-02	alternative ribosomal protein S14	Protein synthesis, modification and degradation			
BSU31590	complement(3246152..3246367)	*yufS*	0.11	3.E-02	putative bacteriocin	Sporulation			
BSU13120	1378496..1379593	*proB*	0.15	1.E-14	glutamate 5-kinase	Amino acid/ nitrogen metabolism			
BSU13130	1379605..1380852	*proA*	0.16	1.E-17	gamma-glutamyl phosphate reductase	Amino acid/ nitrogen metabolism			
BSU32040	complement(3294942..3296270)	*yuiF*	0.17	3.E-13	amino acid transporter	Membrane proteins			
BSU34900	complement(3585690..3586274)	*hisB*	0.18	4.E-03	imidazoleglycerol-phosphate dehydratase	Amino acid/ nitrogen metabolism			
BSU32130	3303042..3304022	*guaC*	0.22	5.E-08	GMP reductase	Nucleotide metabolism			
BSU17470	1880087..1880377	*ynxB*	0.23	6.E-07	putative phage protein	Proteins of unknown function			
BSU01140	134171..135127	*ybaC*	0.23	7.E-07	putative proline iminopeptidase	Poorly characterized/ putative enzymes			
BSU17670	complement(1901117..1901377)	*cotU*	0.24	1.E-01	spore coat protein	Sporulation			
BSU11549	complement(1233133..1233300)	*yizD*	0.24	1.E-04	conserved hypothetical protein	Proteins of unknown function			
BSU17480	1880623..1880967	*ynzF*	0.25	3.E-08	putative phage protein	Proteins of unknown function			
BSU40420	complement(4155433..4156725)	*purA*	0.25	3.E-07	adenylosuccinate synthetase	Nucleotide metabolism			

BSU_misc_RNA_28	1620331..1620445	BSU_misc_RNA_	0.26	2.E-02					
BSU27180	complement(2777877..2778419)	yrhH	0.26	4.E-09	putative methyltransferase	Coping with stress			
BSU20160	complement(2168476..2168871)	yosD	0.27	3.E-02	hypothetical protein; phage SPbeta	Prophages			
BSU34890	complement(3585051..3585689)	hisH	0.28	1.E-02	amidotransferase (glutaminase)	Amino acid/ nitrogen metabolism			
BSU25360	complement(2616667..2616948)	yqfC	0.29	1.E-03	conserved hypothetical protein	Sporulation			
BSU03640	413157..414578	bsdC	0.31	5.E-03	phenolic acid decarboxylase subunit BsdC	Coping with stress			
BSU39990	complement(4107352..4107927)	qdoR	0.31	6.E-05	transcriptional regulator	Regulation of gene expression			
BSU38320	3933209..3933595	cidA	0.32	5.E-05	holin regulator of murein hydrolases	Membrane proteins			
BSU03310	complement(358303..360435)	nasC	0.32	3.E-03	assimilatory nitrate reductase (catalytic subunit)	Amino acid/ nitrogen metabolism			
BSU08350	910019..910651	estB	0.33	3.E-03	secreted esterase / lipase	Lipid metabolism			
BSU25010	complement(2584035..2585327)	yqgE	0.33	2.E-05	putative efflux transporter	Sporulation			
BSU34880	complement(3584317..3585054)	hisA	0.33	2.E-02	phosphoribosylformimino-5-aminoimidazole carboxamide ribotide isomerase	Amino acid/ nitrogen metabolism			
BSU03600	complement(409965..410669)	tcyB	0.33	4.E-08	cystine ABC transporter (permease)	Transporters			
BSU05820	626933..627265	gmuA	0.34	2.E-02	oligo-alpha-mannoside phosphotransferase system enzyme IIA	Transporters			
BSU17260	complement(1861384..1862712)	aprX	0.34	2.E-02	alkaline serine protease	Amino acid/ nitrogen metabolism			
BSU03630	412540..413154	bsdB	0.34	4.E-05	phenolic acid decarboxylase subunit BsdB	Coping with stress			
BSU03610	complement(410656..411462)	tcyA	0.35	5.E-07	cystine ABC transporter (substrate-binding lipoprotein)	Transporters			
BSU36110	3720925..3721401	ywrC	0.35	1.E-04	putative transcriptional regulator (Lrp/AsnC family)	Regulation of gene expression			
BSU38480	complement(3949952..3950584)	relQ	0.35	1.E-06	(p)ppGpp synthetase	Nucleotide metabolism			
BSU06530	710148..711416	purD	0.36	3.E-06	phosphoribosylglycinamide synthetase	Nucleotide metabolism			
BSU34870	complement(3583562..3584320)	hisF	0.37	7.E-03	imidazole glycerol phosphate synthase subunit	Amino acid/ nitrogen metabolism			
BSU06500	706973..708013	purM	0.37	1.E-06	phosphoribosylaminoimidazole synthetase	Nucleotide metabolism			
BSU06520	708594..710132	purH	0.37	1.E-06	fused phosphoribosylaminoimidazole carboxy formyl formyltransferase; inosine-monophosphate cyclohydrolase	Nucleotide metabolism			
BSU20200	complement(2170030..2170242)	yorZ	0.37	2.E-02	hypothetical protein; phage SPbeta	Prophages			
BSU30320	complement(3102629..3105043)	leuS	0.37	3.E-06	leucyl-tRNA synthetase	Protein synthesis, modification and degradation			
BSU33330	complement(3419656..3421065)	lysP	0.37	9.E-04	lysine permease	Amino acid/ nitrogen metabolism			
BSU08990	976569..977033	yhbI	0.38	6.E-05	putative transcriptional regulator (MarR family)	Regulation of gene expression			
BSU06510	708010..708597	purN	0.39	3.E-06	phosphoribosylglycinamide formyltransferase	Nucleotide metabolism			
BSU32410	complement(3327247..3328587)	pucH	0.39	3.E-04	allantoinase	Nucleotide metabolism			
BSU28050	complement(2862572..2863141)	maf	0.39	3.E-06	septum formation DNA-binding protein	Cell envelope and cell division			
BSU18849	complement(2055868..2056107)	yozV	0.39	2.E-02	putative phage protein	Proteins of unknown function			
BSU09520	complement(1029577..1030068)	sigM	0.39	1.E-05	RNA polymerase ECF (extracytoplasmic function)-type sigma factor (sigma(M))	RNA synthesis and degradation			
BSU03590	complement(409208..409951)	tcyC	0.39	3.E-06	cystine ABC transporter (ATP-binding protein)	Transporters			
BSU39029	complement(4006987..4007415)	yxiT_1	0.39	1.E-04	hypothetical protein	Pseudogenes			
BSU36750	complement(3776722..3777753)	spoIID	0.40	6.E-05	autolysin required for complete dissolution of the asymmetric septum (stage II sporulation)	Cell envelope and cell division			
BSU14460	complement(1516339..1516473)	ykpC	0.40	6.E-02	conserved hypothetical protein	Sporulation			
BSU29460	complement(3014514..3015026)	moaB	0.40	8.E-06	molybdopterin GTP-binding precursor Z biosynthesis component	Additional metabolic pathways			
BSU03330	362937..364142	nasA	0.40	3.E-04	putative nitrate transporter	Transporters			
BSU08790	955895..957667	thiC	0.41	2.E-02	biosynthesis of the pyrimidine moiety from 5-aminoimidazole ribotide (AIR)	Additional metabolic pathways			
BSU_misc_RNA_48	complement(2961232..2961479)	tboT	0.41	5.E-04					
BSU03320	complement(360442..362757)	nasB	0.42	2.E-02	assimilatory nitrate reductase (electron transfer subunit NasB)	Amino acid/ nitrogen metabolism			
BSU27260	complement(2786142..2787065)	mccA	0.42	2.E-04	cystathionine beta-synthase for the reverse transsulfuration pathway	Amino acid/ nitrogen metabolism			
BSU17900	1922017..1922463	pcfA	0.42	4.E-05	factor controlling DNA replication	Genetics			
BSU25710	complement(2648903..2649655)	cwlH	0.42	2.E-02	N-acetylmuramoyl-L-alanine amidase	Cell envelope and cell division			
BSU36230	complement(3729488..3730810)	ywqF	0.42	5.E-05	UDP-glucose dehydrogenase	Phosphoproteins			
BSU15490	1620476..1621390	pyrB	0.42	2.E-03	aspartate carbamoyltransferase	Nucleotide metabolism			
BSU18500	complement(2018554..2019270)	yoxD	0.42	2.E-04	putative oxido-reductase	Phosphoproteins			
BSU07260	796314..798233	ltaSA	0.42	2.E-05	exported glycerol phosphate lipoteichoic acid synthetase and anion-binding protein	Cell envelope and cell division			
BSU22610	complement(2369251..2370366)	tyrA	0.42	3.E-05	prephenate dehydrogenase	Amino acid/ nitrogen metabolism			
BSU12900	complement(1357936..1359285)	htrA	0.43	4.E-05	membrane bound serine protease Do, quality control protease (heat-shock protein)	Protein synthesis, modification and degradation			
BSU34860	complement(3582936..3583565)	hisI	0.43	2.E-02	phosphoribosyl-AMP cyclohydrolase; phosphoribosyl-ATP pyrophosphohydrolase	Amino acid/ nitrogen metabolism			
BSU15500	1621374..1622660	pyrC	0.43	5.E-03	dihydroorotase	Nucleotide metabolism			
BSU32990	3383565..3384026	mrgA	0.43	3.E-03	metalloregulation DNA-binding stress protein	Homeostasis			
BSU13740	1440542..1441273	queE	0.43	7.E-04	7-carboxy-7-deazaguanine synthase	Protein synthesis, modification and degradation			
BSU24880	complement(2573520..2573693)	yqgO	0.43	1.E-01	conserved hypothetical protein	Sporulation			
BSU28950	complement(2959257..2961188)	thrS	0.43	2.E-05	threonyl-tRNA synthetase	Protein synthesis, modification and degradation			
BSU23370	2436045..2436917	ypuA	0.43	5.E-05	putative exported protein	Coping with stress			

BSU38330	3933577..3934254	*ywbG*	0.43	3.E-03	anti-holin factor controlling activity of murein hydrolases	Membrane proteins			
BSU24370	complement(2533010..2533699)	*spoIIIAG*	0.44	1.E-01	stage III sporulation engulfment assembly protein	Protein synthesis, modification and degradation			
BSU22310	2340802..2341422	*recU*	0.44	4.E-05	Holliday junction resolvase	Genetics			
BSU12560	1324471..1324980	*xpf*	0.44	4.E-02	putative RNA polymerase PBSX sigma factor-like	RNA synthesis and degradation			
BSU07955	complement(868007..869128)	*yfkA*	0.44	1.E-04	putative Fe-S oxidoreductase, radical SAM superfamily	Protein synthesis, modification and degradation			
BSU16950	1765859..1767034	*pbpX*	0.44	7.E-04	penicillin-binding endopeptidase X	Cell envelope and cell division			
BSU14740	1544603..1544896	*ylaD*	0.44	2.E-02	anti-YlaC sigma factor	Regulation of gene expression			
BSU08170	complement(889372..889686)	*yfjA*	0.44	2.E-03	conserved hypothetical protein	Proteins of unknown function			
BSU14470	complement(1516574..1517581)	*mreBH*	0.44	1.E-03	cell-shape determining protein	Cell envelope and cell division			
BSU26810	2738308..2739105	*yrpC*	0.44	2.E-03	glutamate racemase	Cell envelope and cell division			
BSU27250	complement(2785001..2786140)	*mccB*	0.45	9.E-04	cystathionine gamma-lyase and homocysteine gamma-lyase for reverse transsulfuration pathway	Amino acid/ nitrogen metabolism			
BSU36890	complement(3788426..3789055)	*upp*	0.45	9.E-04	uracil phosphoribosyltransferase	Nucleotide metabolism			
BSU30700	complement(3138978..3139226)	*rpmEB*	0.45	2.E-03	ribosomal protein L31	Protein synthesis, modification and degradation			
BSU30820	complement(3150563..3152305)	*menD*	0.45	2.E-03	2-oxoglutarate decarboxylase and 2-succinyl-5-enolpyruvyl-6-hydroxy-3-cyclohexene-1-carboxylic-acid synthase	Additional metabolic pathways			
BSU06380	696195..696998	*yebC*	0.45	1.E-04	putative integral inner membrane protein	Coping with stress			
BSU37560	complement(3854256..3856172)	*thrZ*	0.45	1.E-02	threonyl-tRNA synthetase	Protein synthesis, modification and degradation			
BSU08610	931879..934464	*yfhO*	0.45	1.E-03	conserved hypothetical protein	Cell envelope and cell division			
BSU13240	complement(1391040..1391642)	*thiU*	0.46	3.E-02	thiamine binding protein	Transporters			
BSU29190	complement(2986588..2987547)	*pfkA*	0.46	2.E-04	6-phosphofructokinase	Carbon metabolism			
BSU27360	complement(2795082..2795735)	*yrrM*	0.46	2.E-03	putative acyl-CoA O-methyltransferase	Poorly characterized/ putative enzymes			
BSU23900	2484911..2485639	*yqjF*	0.46	9.E-03	conserved hypothetical protein	Membrane proteins			
BSU37660	complement(3865355..3866326)	*pta*	0.46	1.E-04	phosphotransacetylase	Carbon metabolism			
BSU30810	complement(3149751..3150575)	*menH_2*	0.46	7.E-03	putative 2-succinyl-6-hydroxy-2,4-cyclohexadiene-1-carboxylate synthase	Additional metabolic pathways			
BSU36240	complement(3731005..3731769)	*ywqE*	0.47	1.E-03	protein tyrosine-phosphatase	Protein synthesis, modification and degradation			
BSU19620	complement(2134566..2135387)	*yodJ*	0.47	3.E-04	D-alanyl-D-alanine carboxypeptidase lipoprotein	Cell envelope and cell division			
BSU23830	2476969..2477730	*yqjL*	0.47	8.E-05	putative hydrolase	Coping with stress			
BSU22300	complement(2339799..2340761)	*yppC*	0.47	2.E-04	conserved hypothetical protein	Sporulation			
BSU06490	705441..706871	*purF*	0.47	2.E-04	glutamine phosphoribosylpyrophosphate amidotransferase	Nucleotide metabolism			
BSU36120	3721415..3722008	*ywrB*	0.47	4.E-03	putative anion transporter	Transporters			
BSU05720	618095..619282	*ydhE*	0.47	2.E-03	putative glycosyltransferase	Coping with stress			
BSU33000	complement(3384070..3385446)	*htrB*	0.47	3.E-04	HtrA-like serine protease	Protein synthesis, modification and degradation			
BSU22430	complement(2353839..2354672)	*panB*	0.47	3.E-04	ketopantoate hydroxymethyltransferase	Additional metabolic pathways			
BSU27820	complement(2843106..2843828)	*yrbC*	0.47	9.E-04	conserved hypothetical protein	Proteins of unknown function			
BSU27560	complement(2816535..2817809)	*hisS*	0.47	1.E-03	histidyl-tRNA synthetase	Protein synthesis, modification and degradation			
BSU33230	complement(3409462..3409992)	*sigO*	0.48	7.E-02	alternative sigma factor	RNA synthesis and degradation			
BSU13720	1439448..1440107	*queC*	0.48	9.E-03	pre-queuosine 0 synthase	Protein synthesis, modification and degradation			
BSU07940	complement(867164..868006)	*yfkC*	0.48	1.E-03	putative mechanosensitive ion channel	Coping with stress			
BSU36250	complement(3731822..3732535)	*ptkA*	0.48	4.E-03	protein tyrosine kinase	Protein synthesis, modification and degradation			
BSU28040	complement(2861840..2862535)	*radC*	0.48	2.E-03	putative DNA repair protein	Genetics			
BSU36260	complement(3732525..3733271)	*ywqC*	0.48	3.E-03	modulator of YwqD protein tyrosine kinase activity	Protein synthesis, modification and degradation			
BSU26840	complement(2742244..2742774)	*sigZ*	0.48	1.E-02	RNA polymerase ECF(extracytoplasmic function)-type sigma factor (sigma-Z)	RNA synthesis and degradation			
BSU30830	complement(3152302..3153717)	*menF*	0.49	7.E-04	menaquinone-specific isochorismate synthase	Additional metabolic pathways			
BSU06550	712019..713293	*yecA*	0.49	1.E-02	putative amino acid/polyamine permease	Transporters			
BSU15550	1628622..1629341	*pyrF*	0.49	5.E-03	orotidine 5'-phosphate decarboxylase	Nucleotide metabolism			
BSU27550	complement(2814743..2816521)	*aspS*	0.49	1.E-03	aspartyl-tRNA synthetase	Protein synthesis, modification and degradation			
BSU22390	complement(2349358..2349528)	*ypmA*	0.49	6.E-03	conserved hypothetical protein	Membrane proteins			
BSU31890	complement(3274462..3275817)	*yukC*	0.49	7.E-03	putative membrane-associated enzyme involved in bacteriocin production	Protein synthesis, modification and degradation			
BSU31080	3187503..3188048	*bslA*	0.49	1.E-03	biofilm hydrophobic layer component	Regulation of gene expression			
BSU12710	1340609..1340875	*xkdR*	0.49	2.E-02	conserved hypothetical protein; putative PBSX prophage protein	Prophages			
BSU09510	complement(1028511..1029587)	*yhdL*	0.49	2.E-04	negative regulator of the activity of sigma-M	Regulation of gene expression			
BSU15480	1619023..1620330	*pyrP*	0.50	4.E-03	uracil permease	Transporters			
BSU34670	complement(3561590..3562183)	*yvdA*	0.50	2.E-02	putative carbonic anhydrase	Poorly characterized/ putative enzymes			
BSU24930	2576367..2576720	*yqzD*	0.50	1.E-02	conserved hypothetical protein	Proteins of unknown function			
BSU00100	17534..18865	*dacA*	0.50	5.E-03	D-alanyl-D-alanine carboxypeptidase (penicillin-binding protein 5)	Cell envelope and cell division			
BSU09000	977069..977734	*yhbJ*	0.50	1.E-02	putative integral membrane protein; putative exporter subunit	Proteins of unknown function			
BSU15510	1622657..1623751	*pyrAA*	0.50	1.E-02	pyrimidine-specific carbamoyl-phosphate synthetase (small subunit, glutaminase subunit)	Nucleotide metabolism			
