# Supplementary material for: Newly Identified Nucleoid-Associated-Like Protein YlxR Regulates Metabolic Gene Expression in Bacillus subtilis
Source: mSphere. 2018 Oct 24;3(5):e00501-18. doi: 10.1128/mSphere.00501-18 (PMC6200986; doi:10.1128/mSphere.00501-18)
Supplement: TABLE S2 [file sph005182669st2.pdf]

Table S2a The list of Spo0A-activated genes and genes that are not driven by Sigma A and SigmaB in the differentially expressed genes in the *ylxR* disruptant with glucose.

	Activated genes in the *ylxR* strain in the presence of glucose	Repressed genes in the *ylxR* strain in the presence of glucose
Spo0A-activated genes (22 genes)	*spoIIE, skfABCEFGH, putBCP, yfmI, spoIIGA-sigE, yodL, spoIIAA-AB-sigF, sdpAB, epr, yxbC*	
Sporulation-related sigmas		
SigE-regulon (22 genes)	*asnO, yjdH, yjfA, yjmD, sigE, yngJ, yodS, yotK, mmgD, yqfZ, yqxA, nucB, ysnD, glgBCDAP, spoIIID, yybI*	*spoIII-AG, spoIID*
SigF-regulon (15genes)	*yabT, yfhD, yfiL, yfiN, yjbA, pbpG, spoIIGA-sigE, spoIIAA-AB-sigF, spoIVB, yqxA, ytfJ,*	*yqfC*
SigK-regulon (13 genes)	*ydgA, cotA, yfkT, gerPF, yitBA, ymaG, yoaN, yotK, ysnD, ytlD, ytxO*	*cwlH, cotU*
SigG-regulon (11 genes)	*ydfS, yfkR, yfhD, yfiL-yfiN, pbpG, spoIIGA-sigE, spoIIAA-AB-sigF,*	
ECF sigmas		
SigX-regyulon (3 genes)		*yfhO, pbpX, yrhH*
SigM-regulon (8 genes)		*yebC, pbpX, maf, ypuA, yqjL, yrhH, recU, sigM*
Other sigmas		
SigI-regulon (2 genes)		*mreBH-ykpC*
SigO-reguron (1 genes)		*sigO*
SigD-regulon (23 genes)	*ybdO, hemAT, yjcQ, yjfB, mcpC, cheV,ylqGH, ylqB(sivC), yoyG, yqaR, yqaS, yscB, lytB, mcpB-tlpA-mcpA-tlpB, yvaQ, yvyFG, epr, yxkC*	
SigL-regulon (6 genes)	*ptb-bcd-buk, rocBC, rocF(argI)*	

Table S2b. The list of genes involved in synthesis of co-factor in the differentially expressed genes in the *ylxR* disruptant with glucose.

Expression; down	Co-factor
thiC	Thiamine
menD	Menaquinon
menH	Menaquinon
panB	Co-Enzyme A
moaB	Molybdopterin
