# Supplementary material for: Newly Identified Nucleoid-Associated-Like Protein YlxR Regulates Metabolic Gene Expression in Bacillus subtilis
Source: mSphere. 2018 Oct 24;3(5):e00501-18. doi: 10.1128/mSphere.00501-18 (PMC6200986; doi:10.1128/mSphere.00501-18)
Supplement: FIG S4 [file sph005182669sf4.pdf]

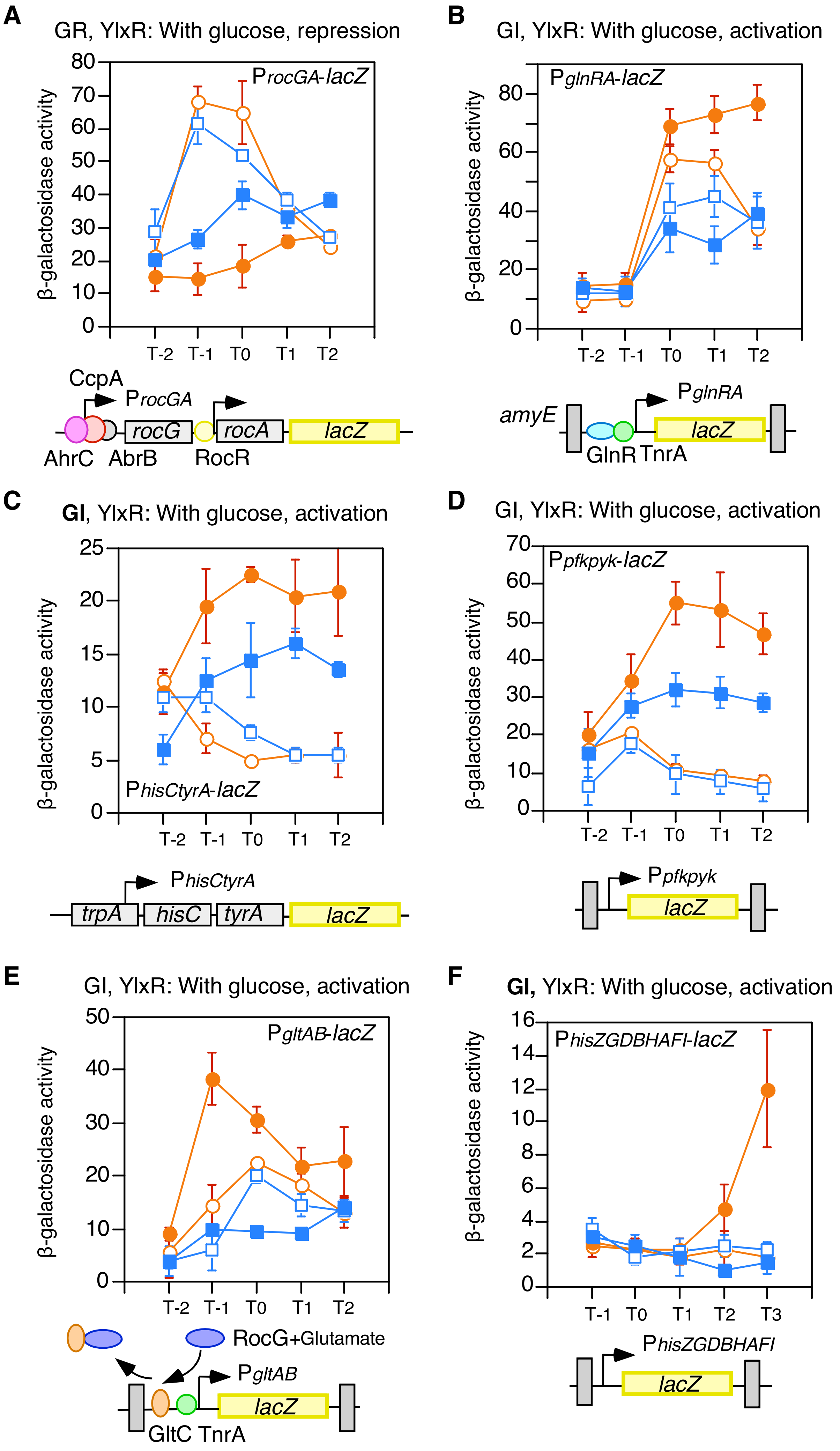

Fig. S4-continued

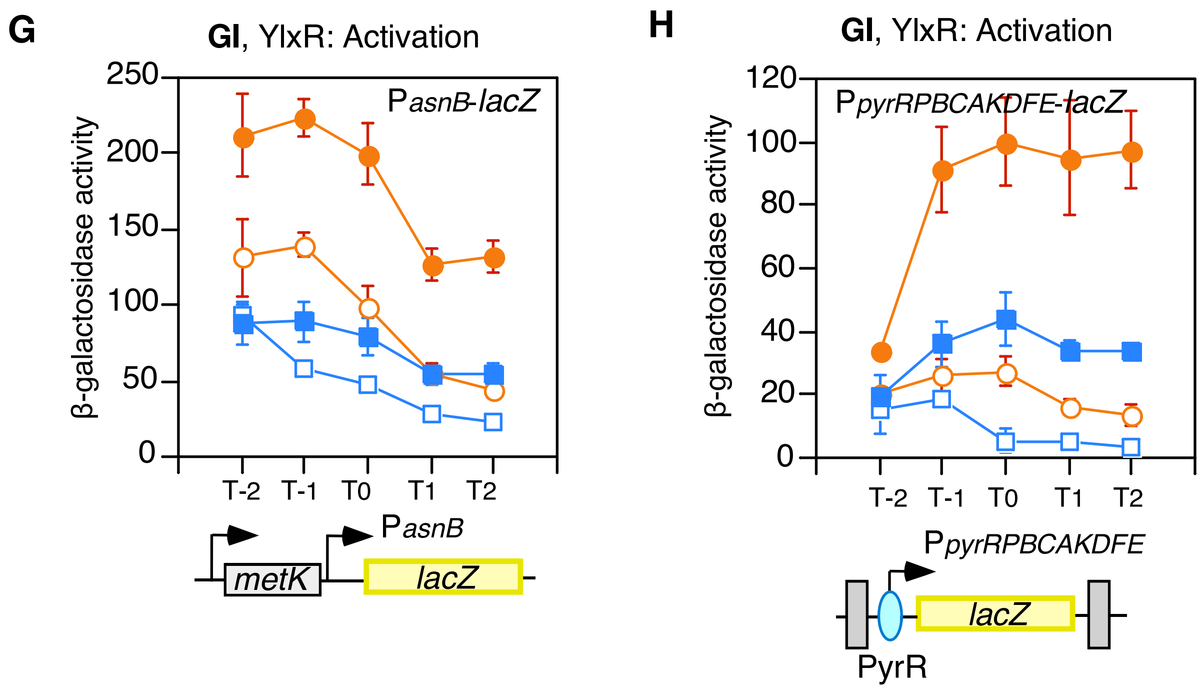

G
GI, YlxR: Activation
250
200
150
100
50
0
β-galactosidase activity
PasnB-lacZ
T-2 T-1 T0 T1 T2
PasnB
metK
lacZ
H
GI, YlxR: Activation
120
100
80
60
40
20
0
β-galactosidase activity
PpyrRPBCAKDFE-lacZ
T-2 T-1 T0 T1 T2
PpyrRPBCAKDFE
lacZ
PyrR

Fig. S4
