# Supplementary material for: Newly Identified Nucleoid-Associated-Like Protein YlxR Regulates Metabolic Gene Expression in Bacillus subtilis
Source: mSphere. 2018 Oct 24;3(5):e00501-18. doi: 10.1128/mSphere.00501-18 (PMC6200986; doi:10.1128/mSphere.00501-18)
Supplement: TABLE S3 [file sph005182669st3.pdf]

Table S3. Strains and plasmids used in the Supplementary section of this study.

Strain	Genotype	Reference or source
OAM741	*trpC2 thrC*::P*ylxS-lacZ* (-284/+77[1] Sp^{r})	This study
501-77	*trpC2 pdhC* (Km^{r})	(1)
OAM735	*trpC2 ylxR* (Em^{r} *lacZ*::Tc^{r})	This study
OAM769	*trpC2 thrC*::P*ylxS-lacZ* (Sp^{r}) *pdhC* (Km^{r})	This study
OAM725	*trpC2 thrC::sigX-lacZ* (Em^{r}) *cshA* (Tc^{r}) *bkdB*::P*xyl-cshA* (Km^{r})	(2)
OAM727	*trpC2 thrC*::*sigX-lacZ* (Em^{r}) *cshA* (Tc^{r}) *bkdB*::P*xyl-cshA* (K244R, K296R, Km^{r})	(2)
OAM743	*trpC2 thrC*::P*ylxS-lacZ* (-284/+77[1] Sp^{r}) *cshA* (Tc^{r}) *bkdB*::P*xyl-cshA* (Km^{r})	This study
OAM814	*trpC2 thrC*::P*ylxS-lacZ* (-284/+77[1] Sp^{r}) *cshA* (Tc^{r}) *bkdB*::P*xyl-cshA* (K244R, K296R, Km^{r})	This study
OAM818	*trpC2 amyE*::P*ylxS-gfp* (-284/+77[1] Cm^{r})	This study
OAM819	*trpC2 rocA*::pMutin-rocA (Em^{r})	This study
OAM851	*trpC2 rocA*::pMutin-rocA (Em^{r}) *ylxR* (Km^{r})	This study
OAM850	*trpC2 amyE*::P*glnR-lacZ* (-255 /+24[1], Cm^{r})	This study
OAM831	*trpC2 amyE*::P*glnR-lacZ* (-255 /+24[1], Cm^{r}) *ylxR* (Km^{r})	This study
OAM832	*trpC2 tyrA*::pMutin-tyrA (Em^{r})	This study
OAM833	*trpC2 tyrA*::pMutin-tyrA (Em^{r}) *ylxR* (Km^{r})	This study
OAM834	*trpC2 amyE*::P*pfk-lacZ* (-323 /+1[2], Cm^{r})	This study
OAM835	*trpC2 amyE*::P*pfk-lacZ* (-323 /+1[2], Cm^{r}) *ylxR* (Km^{r})	This study
OAM827	*trpC2 amyE*::P*gltA-lacZ* (-326 /+56[1], Cm^{r})	This study
OAM828	*trpC2 amyE*::P*gltA-lacZ* (-326 /+56[1], Cm^{r}) *ylxR* (Km^{r})	This study
OAM838	*trpC2 amyE*::P*hisZ-lacZ* (-296 /+1[2], Cm^{r})	This study
OAM839	*trpC2 amyE*::P*hisZ-lacZ* (-296 /+1[2], Cm^{r}) *ylxR* (Km^{r})	This study
BFS56	*trpC2 asnB*::pASNB5D (Em^{r})	(3)
OAM823	*trpC2 asnB*::pASNB5D (Em^{r}) *ylxR* (Km^{r})	This study
OAM825	*trpC2 amyE*::P*pyrR-lacZ* (-250 /+150[1], Cm^{r})	This study
OAM826	*trpC2 amyE*::P*pyrR-lacZ* (-250 /+150[1], Cm^{r}) *ylxR* (Km^{r})	This study
**Plasmid**	**Description**	
pIS284	Insertion vector to *amyE*, chloramphenicol resistance, *lacZ*	(4)
pIS-glnR	pIS284 carrying a promoter region of *glnR*	This study
pIS-pfk	pIS284 carrying a promoter region of *pfk*	This study
pIS-gltA	pIS284 carrying a promoter region of *gltA*	This study
pIS-hisZ	pIS284 carrying a promoter region of *hisZ*	This study
pIS-pyrR	pIS284 carrying a promoter region of *pyrR*	This study
pMutin2	Insertion vector, Ampicillin and erythromycin resistance, *lacZI*	(3)
pMutin-rocA	pMutin2 carrying a part of *rocA*	This study
pMutin-tyrA	pMutin2 carrying a part of *tyrA*	This study

[1] Numbers indicate the nucleotide positions relative to the transcription start point.

[2] Numbers indicate the nucleotide positions relative to the translation start point.

1. Gao H, Jiang X, Pogliano K, Aronson AI. 2002. J Bacteriol 184:2780-2788.
2. Ogura M, Asai K. 2016. Front Microbiol 7:1918.
3. Vagner V, Dervyn E, Ehrlich SD. 1998. Microbiology 144:3097-3104.2016. Front Microbiol 7:1918.
4. Tsukahara K, Ogura M. 2008. BMC Microbiol. 8:8.
