# Supplementary material for: Newly Identified Nucleoid-Associated-Like Protein YlxR Regulates Metabolic Gene Expression in Bacillus subtilis
Source: mSphere. 2018 Oct 24;3(5):e00501-18. doi: 10.1128/mSphere.00501-18 (PMC6200986; doi:10.1128/mSphere.00501-18)
Supplement: TABLE S4 [file sph005182669st4.pdf]

Table S4. Oligonucleotides used in this study.

Name	Sequence	Product/use
pX-ylxR-Spe	5'-AAACTAGTTGAATAAGAGGTGACTTTAGGTG-3'	pX-ylxR
pX-ylxR-Bam	5'-ATGGGATCCTTATTTTTTCACCTTTTCCGCCAGTTCC-3'	pX-ylxR
ylxR-Eco	5'-AATGAATTCGCCTGATCAAAACCAATTGTCACTG-3'	pDG1729-ylxS
ylxR-Hin	5'-GCTAAGCTTGTTCTTCCTCCTTGTGCTGTCG-3'	pDG1729-ylxS
ylxR-chitin-F (Sap)	5'-GGTTGCTCTTCAAACGTGAATAAACACAAAAAGATCCC-3'	pTYB11-ylxR
ylxR-chitin-R (Xh)	5'-TTGCTCGAGTTATTTTTTCACCTTTTCCGCCAGTTCC-3'	pTYB11-ylxR
amyE-RR	5'-GTTAACAAAATTCTCCAGTCTTCACATCGG-3'	OAM818
amyE-FF	5'-TCAATGGGGAAGAGAACCGCTTAAGCCCG-3'	OAM818
gfp(SD)-F	5'-CATAAGGAGGAACTACTATGAGTAAAG-3'	OAM818
PylxR-(SD)-gfp-R	5-GTAGTTCCTCCTTATGGTTCTTCCTCCTTGTGCTGTCG -3	OAM818
ylxQ-FF	5'-GCTGTTCATATCGACCGCGACAACTGTTATTGTTCCTG-3'	ylxQ (Tc^r)
ylxQ-FR	5'-TGGCCAGACCCAGCAAGGGAA-3'	ylxQ (Tc^r)
ylxQ-RF	5'-TTTTTTTATAACAGGAATTCCTGACCAAGGTTTTGCGAATA-3'	ylxQ (Tc^r)
ylxQ-RR	5'-AGGCTTTTGCTGATGTTGGTTTT-3'	ylxQ (Tc^r)
TC-F	5'-GGTCGATATGAACAGCTTATTTAC-3'	ylxQ (Tc^r)
TC-R	5'-GAATTCCTGTTATAAAAAAAGGATCAA-3'	ylxQ (Tc^r)
ylxR-gfp-F	5- ATCGGATCCGAGAGAACTTGAAGAAGA-3	pSG1194-ylxR
ylxR-gfp-R	5-CTTCTCCTTTACTCATTTTTTTCACCTTTTCCGCCAG-3	pSG1194-ylxR
gfp-F	5'-ATGAGTAAAGGAGAAGAACTTTTCAC-3'	pSG1194-ylxR
gfp-Xba-R	5'-GCTCTAGATTATTTGTATAGTTCATCCATGCCA-3'	pSG1194-ylxR
yqfO-E	5'-AATGAATTCTGGATGATATGGCAGAGTGG-3'	pIS-trmK
pIS-trmK-B	5'-ATCGGATCCACCGCTCCATTCGGTATGT-3'	pIS-trmK
pIS-gltA-E	5-ATTGAATTCGCATAATCAATGGCTTTC-3	pIS-gltA
pIS-gltA-B	5-TTCGGATCCAATTCCTCTCCCCCGATCAA-3	pIS-gltA
pIS-pyrR-F(E)	5-ATTGAATTCGAACCCATCAAATTCGTGTTC-3	pIS-pyrR
pIS-pyrR-R(B)	5-TTCGGATCCTGTGTGACACCTCACAGTTCAT-3	pIS-pyrR
Mut-rocA-E	5-ATTGAATTCGATCTTTGCAGATGTTGATGAA-3	pMuint-rocA
Mut-rocA-B	5-TTCGGATCCTCAAAAAGCTTCGGACGTTGTTTT-3	pMuint-rocA
pIS-glnR-E	5-ATTGAATTCATTATGGCAGCAGGGACG-3	pIS-glnR
pIS-glnR-B	5-TTCGGATCCTCAATTTCCTCCTTTTCTTAAC-3	pIS-glnR
pIS-pfk-F(E)	5-ATCGAATTCGCTATATGGACGAAACCCTTAA-3	pIS-pfk
pIS-pfk-F(B)	5-ATCGGATCCTCTCCATTCACCTCAGCAAC-3	pIS-pfk
pIS-hisZ-E	5-ATTGAATTCGCCTGAAAGAATCATCAGG-3	pIS-hisZ
pIS-hisZ-B	5-TTCGGATCCAACCCGAGCACCTCCGC	pIS-hisZ
tyrA-Mut-E	5-GTTGAATTCTTAGACCGTTTTGATGAGTG-3	pMuint-tyrA
tyrA-Mut-B	5-TTCGGATCCTCAATCAGCATAAAAAGTTTCATATT-3	pMuint-tyrA
