# Supplementary material for: Newly Identified Nucleoid-Associated-Like Protein YlxR Regulates Metabolic Gene Expression in Bacillus subtilis
Source: mSphere. 2018 Oct 24;3(5):e00501-18. doi: 10.1128/mSphere.00501-18 (PMC6200986; doi:10.1128/mSphere.00501-18)
Supplement: TABLE S5 [file sph005182669st5.pdf]

Ttable S5. Numbers of reads obtained by NGS.

Sample name	description	Number of original reads	Number of trimmed reads	Numbr of reads mapped to the reference
W_1	1st test read1: wild-type cells	7,201,299	6,321,053	6,242,098
W_2	1st test read2: wild-type cells	7,201,299	5,823,012	5,738,073
R_1	1st test read1: *ylxR* mutant cells	4,513,546	3,829,743	3,755,815
R_2	1st test read2: *ylxR* mutant cells	4,513,546	3,490,437	3,418,067
W2_1	2nd test read1: wild-type cells	5,480,907	4,849,922	4,788,937
W2_2	2nd test read2: wild-type cells	5,480,907	4,595,317	4,535,958
R2_1	2nd test read1: *ylxR* mutant cells	4,990,445	4,329,389	4,164,122
R2_2	2nd test read2: *ylxR* mutant cells	4,990,445	4,311,328	4,142,047
W3_1	3rd test read1: wild-type cells	4,583,775	4,054,658	4,006,598
W3_2	3rd test read2: wild-type cells	4,583,775	3,884,830	3,837,736
R3_1	3rd test read1: *ylxR* mutant cells	6,344,344	5,544,533	3,609,076
R3_2	3rd test read2: *ylxR* mutant cells	6,344,344	5,401,946	3,560,476

Parameters for quality trimming was as follows. Parameters; Phred quality score >30; Removing terminal 20 nucleotides from 5'-end and 5 nucleotides from 3'-end; removing truncated reads less than 50-nucleotides length.

Trimmed reads were mapped to the all genes in Bacillus subtilis 168 (accession number: AL009126.3) using CLC Genomics Workbench ver. 10.0.1. (Qiagen) with following parameters; Length fraction: 0.8; Similarity fraction: 0.9; Maximum number of hits for a read: 1. The numbers of original reads, quality trimmed reads and mapped reads were listed.
